# Extracorporeal Membrane Oxygenation for Pulmonary Embolism: A Systematic Review and Meta-Analysis

**DOI:** 10.3390/jcm13010064

**Published:** 2023-12-22

**Authors:** Jonathan Jia En Boey, Ujwal Dhundi, Ryan Ruiyang Ling, John Keong Chiew, Nicole Chui-Jiet Fong, Ying Chen, Lukas Hobohm, Priya Nair, Roberto Lorusso, Graeme MacLaren, Kollengode Ramanathan

**Affiliations:** 1 Faculty of Medicine, University of New South Wales, Sydney, NSW 2052, Australia; 2 South Western Sydney Clinical Campuses, University of New South Wales, Sydney, NSW 2170, Australia; 3 Cardiothoracic Intensive Care Unit, National University Heart Centre, National University Hospital, Singapore 119074, Singapore; 4 Yong Loo Lin School of Medicine, National University of Singapore, National University Health System, Singapore 119228, Singapore; 5 Royal College of Surgeons in Ireland (RCSI), University College Dublin (UCD) Malaysia Campus, D02 YN77 Dublin, Ireland; 6 Agency for Science, Technology and Research (A*STaR), Singapore 138632, Singapore; 7 Department of Cardiology, Cardiology I and Center for Thrombosis and Hemostasis (CTH), University Medical Center Mainz, 55131 Mainz, Germany; 8 Department of Intensive Care, St. Vincent’s Hospital Sydney, Darlinghurst, NSW 2010, Australia; 9 Cardio-Thoracic Surgery Department, Heart and Vascular Centre, Maastricht University Medical Centre, 6229 HX Maastricht, The Netherlands; 10 Cardiovascular Research Institute Maastricht, 6229 ER Maastricht, The Netherlands

**Keywords:** extracorporeal membrane oxygenation, pulmonary embolism, mortality, meta-analysis

## Abstract

Background: The use of extracorporeal membrane oxygenation (ECMO) for high-risk pulmonary embolism (HRPE) with haemodynamic instability or profound cardiogenic shock has been reported. Guidelines currently support the use of ECMO only in patients with cardiac arrest or circulatory collapse and in conjunction with other curative therapies. We aimed to characterise the mortality of adults with HRPE treated with ECMO, identify factors associated with mortality, and compare different adjunct curative therapies. Methods: We conducted a systematic review and meta-analysis, searching four international databases from their inception until 25 June 2023 for studies reporting on more than five patients receiving ECMO for HRPE. Random-effects meta-analyses were conducted. The primary outcome was in-hospital mortality. A subgroup analysis investigating the outcomes with curative treatment for HRPE was also performed. The intra-study risk of bias and the certainty of evidence were also assessed. This study was registered with PROSPERO (CRD42022297518). Results: A total of 39 observational studies involving 6409 patients receiving ECMO for HRPE were included in the meta-analysis. The pooled mortality was 42.8% (95% confidence interval [CI]: 37.2% to 48.7%, moderate certainty). Patients treated with ECMO and catheter-directed therapy (28.6%) had significantly lower mortality (*p* < 0.0001) compared to those treated with ECMO and systemic thrombolysis (57.0%). Cardiac arrest prior to ECMO initiation (regression coefficient [B]: 1.77, 95%-CI: 0.29 to 3.25, *p* = 0.018) and pre-ECMO heart rate (B: −0.076, 95%-CI: −0.12 to 0.035, *p* = 0.0003) were significantly associated with mortality. The pooled risk ratio when comparing mortality between patients on ECMO and those not on ECMO was 1.51 (95%-CI: 1.07 to 2.14, *p* < 0.01) in favour of ECMO. The pooled mortality was 55.2% (95%-CI: 47.7% to 62.6%), using trim-and-fill analysis to account for the significant publication bias. Conclusions: More than 50% of patients receiving ECMO for HRPE survive. While outcomes may vary based on the curative therapy used, early ECMO should be considered as a stabilising measure when treating patients with HRPE. Patients treated concurrently with systemic thrombolysis have higher mortality than those receiving ECMO alone or with other curative therapies, particularly catheter-directed therapies. Further studies are required to explore ECMO vs. non-ECMO therapies in view of currently heterogenous datasets.

## 1. Introduction

Acute pulmonary embolism (PE) is a significant cause of shock or cardiac arrest and can be associated with high mortality rates. In the United States and Europe, PE accounts for approximately 100,000 and 300,000 annual deaths, respectively [1,2]. High-risk pulmonary embolisms (HRPE) are emergencies that result in haemodynamic instability, refractory cardiogenic shock, or cardiac arrest. Up to 5% of PEs are high-risk and have variable survival rates, ranging from 15% to 65% despite emergency treatment [3,4]. Guidelines currently support the use of extracorporeal membrane oxygenation (ECMO) only in patients with cardiac arrest or circulatory collapse and in conjunction with other curative therapies, albeit with a low level of recommendation [5].

ECMO has been used as a cardiopulmonary haemodynamic support for stabilising patients presenting with haemodynamic instability or refractory cardio-circulatory compromise secondary to HRPE [6,7]. ECMO has been successfully utilised in HRPE management as a bridge to definitive treatment, as well as a bridge to recovery following treatment, and by itself as a stabilising intervention without curative therapy other than anticoagulation [8,9,10]. Despite this, ECMO is an invasive and resource-intensive form of life support with a high rate of potential complications [11,12]. Previous reviews on this topic were based on a smaller cohort of patients from publications up to 2020 [6,13,14,15]. Thus, we sought to provide updated knowledge regarding the use of ECMO in HRPE, its outcomes, and predictors regarding mortality.

## 2. Methods

### 2.1. Search Strategy and Selection Criteria

This study was registered on PROSPERO (CRD42022297518) and conducted in adherence with the Preferred Reporting Items for Systematic reviews and Meta-Analyses statement (Table S1) [16]. We searched MEDLINE via PubMed, EMBASE via Ovid, Cochrane Library, and Scopus using the keywords “extracorporeal membrane oxygenation” and “pulmonary embolism” from 1 January 1962 to 25 June 2023 (Table S2). We also reviewed the reference lists of included studies and review articles. We included studies reporting on the use of ECMO for PE with >5 patients. We excluded studies detailing ECMO use non-specific to PE, animal models and studies, correspondences, reviews, non-English publications, studies without a full text, and studies including chronic thromboembolic disease. In the case of overlapping patient data, we included the largest study without compromising on granular data and excluded all other studies.

### 2.2. Data Collection and Risk of Bias Assessment

Data were collected using a prespecified data extraction form (Table S3). Where necessary, we contacted study authors for additional data or clarification regarding the data reported. The intra-study risk of bias was rated using the appropriate Joanna Briggs Institute checklist [17]. The overall certainty of evidence was assessed using the Grading of Recommendations, Assessment, Development, and Evaluations (GRADE) approach. The screening of studies, data collection, and risk of bias assessment were conducted independently in duplicate by JJEB, RRL, JKC, and NF; conflicts were resolved by consensus or by KR. As per the GRADE guidelines, we applied informative statements to communicate the certainty of the pooled estimate [18]. Overall high certainty of the level of evidence is termed as “the outcome is”; moderate certainty has been phrased as “the outcome is probably”; we summarise any case of low certainty as “the outcome may be”; and very low certainty outcomes have been interpreted as “we are uncertain if the outcome is”. Curative therapy was grouped into surgical embolectomy, catheter-directed therapy (which consists of catheter-directed embolectomy and thrombolysis), and systemic thrombolysis.

### 2.3. Data Synthesis

The primary outcome was in-hospital mortality; 30-day mortality was utilised where in-hospital mortality was not reported. Secondary outcomes included the duration of ECMO, the lengths of ICU and hospital stays, and complications while on ECMO. Statistical analyses were performed using R4.1.3. Random-effects meta-analyses (DerSimonian and Laird) were conducted based on the logit transformation, and 95% confidence intervals (CIs) were computed using the Clopper–Pearson method [19,20,21,22]. Dichotomous outcomes are presented as pooled proportions or pooled risk ratios (RR) if a comparator group is present, and continuous outcomes as pooled means or mean differences if a comparator group is present, each with their corresponding 95% confidence intervals (CIs). For comparative meta-analyses, we used a continuity correction factor of 0.5 to include studies with zero events. We conducted several sensitivity analyses. First, we excluded any studies with high risks of bias or with a sample size ≤ 10. Secondly, we carried out a post hoc analysis limiting our analysis to patients receiving only VA-ECMO. Publication bias was assessed using Egger’s test or visual inspection of the funnel plots if fewer than ten studies were included. A factor of 0.5 was used for continuity correction.

A pre-specified subgroup analysis was conducted based on curative therapy received. Post hoc subgroup analyses included region, study type, venoarterial-ECMO (VA-ECMO) vs. venovenous-ECMO (VV-ECMO), and duration of follow-up. Univariable meta-regression was conducted when at least six data points were reported to investigate potential sources of heterogeneity and prognostically relevant study-level covariates [12]. Where the means and standard deviations were not available, we derived them from the data presented in each study by Wan and colleagues [23]. As inter-study heterogeneity can be overestimated by I^2^ statistics, we assessed the inter-study heterogeneity using GRADE [24,25]. A *p*-value of <0.05 was considered significant in our analysis. Specifically, when comparing mortality rates between individual subgroups in a pairwise fashion, we applied a Bonferroni correction as appropriate (given seven pairwise comparisons, the appropriate *p*-value would be 0.05/7 = 0.0071).

### 2.4. Role of the Funding Source

There was no funding source for this study.

## 3. Results

**Figure 1:**
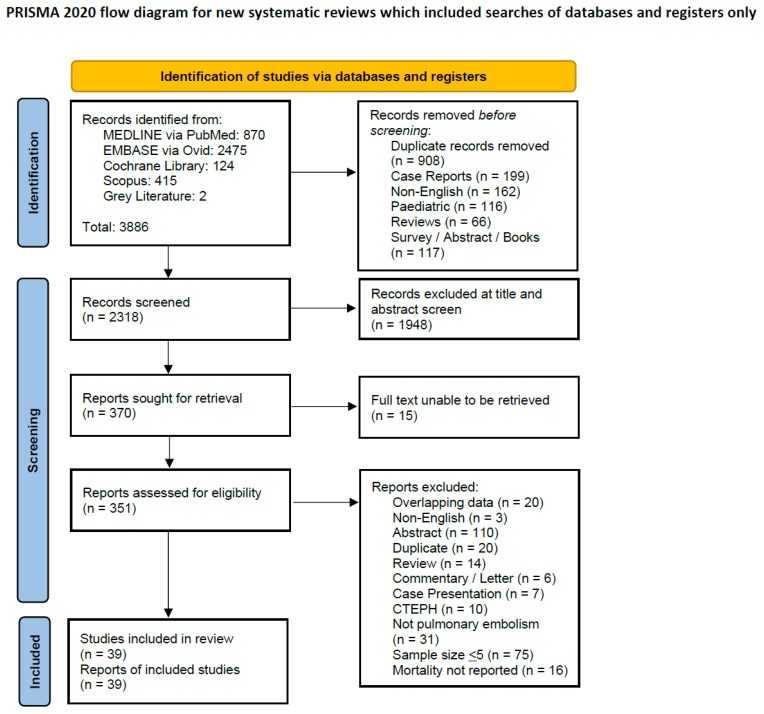
Preferred Reporting Items for Systematic reviews and Meta-Analyses 2020 flow diagram [16].

Of 2318 references screened, we sought the full text for 370 potentially relevant publications. Some 351 full-text articles were available, from which we included 39 observational studies totalling 1,177,998 patients (6409 ECMO and 1,171,589 non-ECMO) in the meta-analysis (Figure 1, Table S4) [9,10,26,27,28,29,30,31,32,33,34,35,36,37,38,39,40,41,42,43,44,45,46,47,48,49,50,51,52,53,54,55,56,57,58,59,60,61,62], of which 13 were cohort studies and 26 were case series. There were 14 studies from Europe, 14 from Asia, and 11 from North America. One study had a control group of 1,170,157 patients and was not limited to patients with HRPE [48]. There was a higher proportion of males 52.5% (95%-CI: 47.8% to 57.1%) receiving ECMO, with a mean age of 53.5 years (95%-CI: 51.7% to 55.3%). The pooled pre-ECMO pH (16 studies, 456 patients) was 7.10 (95%-CI: 7.05 to 7.16), while the pooled pre-ECMO lactate (14 studies, 503 patients) was 9.35 mmol/L (95%-CI: 7.64 to 11.07). The pooled time to ECMO from the onset of shock was 3.44 h (95%-CI: −2.08 to 8.97), while ECMO was initiated within 24.6 min (95%-CI: 1.2 to 48.0) in patients who had cardiac arrest. Table S4 summarises the study characteristics, patient demographics and outcomes. Table S5 summarises the intra-study risk of bias, and Table S6 details the GRADE assessment; all the studies were of good quality (JBI score > 6). We further explored the demographics of the patients recruited in each region (Asia, North America, and Europe). Table S4 summarises the demographics by region. We approached one author to clarify the data [48]. No unpublished data were provided.

**Figure 2:**
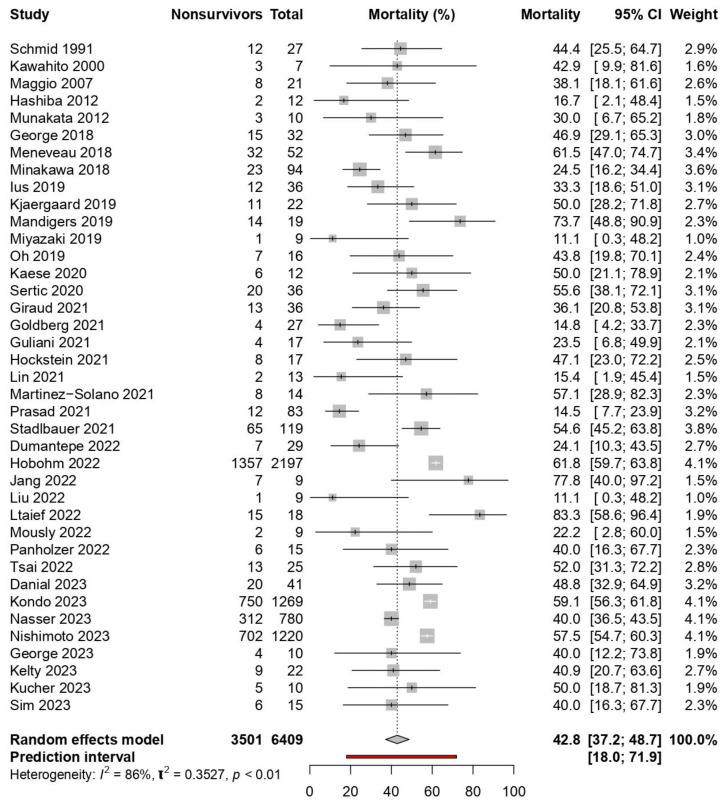
Pooled mortality for patients receiving extracorporeal membrane oxygenation for high-risk pulmonary embolism [9,10,26,27,28,29,30,31,32,33,34,35,36,37,38,39,40,41,42,43,44,45,46,47,48,49,50,51,52,53,54,55,56,57,58,59,60,61,62].

**Figure 3:**
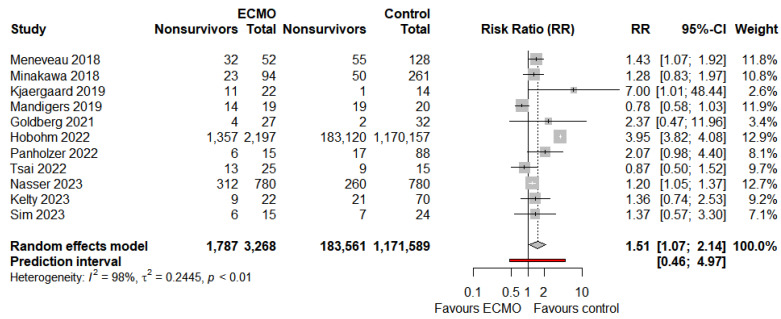
Pooled risk ratio comparing patients receiving ECMO vs. no ECMO for high-risk pulmonary embolism [29,31,37,38,40,41,46,48,58,60,62].

The pooled mortality for patients receiving ECMO for HRPE was 42.8% (39 studies, 95%-CI: 37.2% to 48.7%, p_egger_ < 0.0001, moderate certainty, Figure 2). Sensitivity analyses excluding studies with ≤10 patients (6 studies, n = 33) did not change mortality substantially (33 studies, 43.4%, 95%-CI: 37.4% to 49.6%). In view of the significant publication bias, we conducted a random-effects trim-and-fill analysis (R_0_ estimator) [63] to explore the potential result if the studies contributing to the publication bias were nullified; 15 studies were added, and the pooled mortality was 55.2% (95%-CI: 47.7% to 62.6%). Figure S1 includes the trim-and-fill analysis superimposed over a funnel plot for mortality in patients receiving ECMO. We compared the mortality rates of patients receiving and not receiving ECMO; this was statistically significant (11 studies, RR 1.51: 95%-CI: 1.07 to 2.14, *p* = 0.0198, Figure 3). Since one study had a control group of patients not limited to HRPE, we conducted a sensitivity analysis excluding this study [48]. This reduced the pooled RR to 1.21 and rendered the result statistically insignificant (10 studies, 95%-CI: 0.99 to 1.49, *p* = 0.0664, Figure S2). Two other sensitivity analyses were also conducted. First, we excluded a study [48] accounting for a large number of ECMO patients (n = 2143)—the mortality rate remained relatively unchanged (42.1%, 95%-CI: 36.4% to 48.0%). Secondly, we restricted the analysis to studies reporting on only VA-ECMO—the mortality rate also remained relatively unchanged (41.1%, 95%-CI: 35.0% to 47.5%).

**Figure 4:**
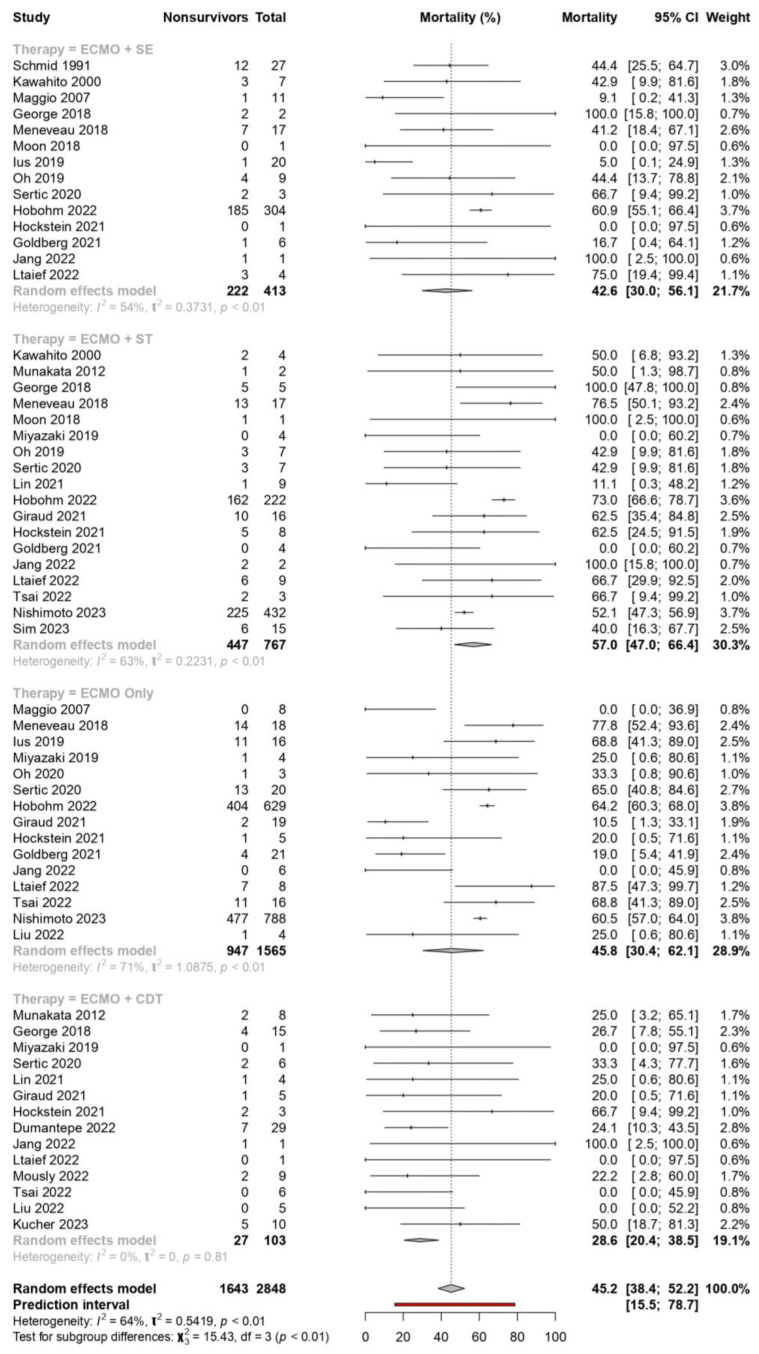
Subgroup analysis of mortality for patients receiving extracorporeal membrane oxygenation alone compared to ECMO with catheter-directed therapies (CDT), surgical embolectomy (SE), or systemic thrombolysis (ST) for pulmonary embolism [9,10,26,27,28,29,30,31,32,33,34,35,36,37,38,39,40,41,42,43,44,45,46,47,48,49,50,51,52,53,54,55,56,57,58,59,60,61,62].

We explored the mortality of patients receiving individual types of curative therapy concurrently with ECMO, and we found significant differences (*p* = 0.0015, Figure 4). A strategy of spontaneous recovery with ECMO alone (45.81%) had a higher mortality rate than ECMO with concurrent surgical embolectomy (42.6%) or catheter-directed therapies (28.6%). Patients receiving systemic thrombolysis had the highest mortality rates (56.99%). We conducted a test for subgroup differences between individual therapies, which showed that mortality after systemic thrombolysis compared to catheter-directed therapies was statistically significant after Bonferroni correction (*p* < 0.0001, Figure 4). Catheter-directed therapies when compared against ECMO alone (*p* = 0.034) and surgical embolectomy (*p* = 0.043) were not statistically significant after a Bonferroni correction (p_threshold_ = 0.0071). The test for subgroup differences between other individual therapies was otherwise statistically insignificant. Table S7 summarises the above. Five studies (93 patients) reported on catheter-directed thrombolysis, while three studies (8 patients) reported on catheter-directed embolectomy (Table S7).

We found that mortality varied when stratifying studies based on geographical regions (*p* = 0.0071, Figure S3). Studies reporting on patients from European centres reported the highest mortality (53.1%, 95%-CI: 46.3% to 59.8%), followed by those from Asia (37.8%, 95%-CI: 27.6% to 49.1%) and those from North America (34.5%, 95%-CI: 25.6% to 44.6%). The demographics for each regional subgroup by age, proportion of males, pre-ECMO pH, and pre-ECMO lactate are further shown in Table S4. There was no significant difference in mortality when the results were stratified by study type (*p* = 0.95, Figure S4). Fewer than six studies reported on VA-ECMO vs. VV-ECMO and hence an analysis wasomitted.

Univariable meta-regression analysis found that the proportion of patients who suffered from cardiac arrest either before or during ECMO (regression coefficient [B]: 1.77, 95%-CI: 0.29 to 3.25, *p* = 0.018) and pre-ECMO heart rate (B: −0.076, 95%-CI: −0.12 to −0.035, *p* = 0.0003) were significantly associated with mortality. Other covariates including malignancy, hypertension, time to ECMO, and duration of ECMO were not significantly associated with mortality. Details of the meta-regression analyses are presented in Table S7.

### 3.1. Secondary Outcomes

The pooled duration of ECMO was 4.4 days (95%-CI: 3.6 to 5.1, moderate certainty, Figure S5). The ICU length of stay was 12.6 days (95%-CI: 9.7 to 15.4, moderate certainty, Figure S6), and the hospital length of stay was 22.5 days (95%-CI: 18.1 to 26.9, moderate certainty, Figure S7). The pooled duration of mechanical ventilation was 7.2 days (95%-CI: 2.7 to 11.6, moderate certainty, Figure S8). Details of the secondary outcomes are summarised in Table S8. Some 32 studies (4841 patients) reported a total of 2454 complications during ECMO, of which non-septic shock, acute kidney injury, and intracranial haemorrhage were the most common (Table S9). Additionally, the pooled incidence of haemorrhagic complications was 35.9% (95%-CI: 25.5% to 47.8%, Figure S9).

### 3.2. Post Hoc Analysis

We conducted a post hoc meta-analysis on each study’s reported follow-up duration. Our meta-analysis on mortality was based on the earliest measure of mortality in studies, which was either in-hospital mortality (39.5%, 95%-CI: 33.2% to 46.0%, 33 studies) or 30-day mortality (51.2%, 95%-CI: 38.8% to 63.4%, 14 studies). Three studies reported 60-day mortality (46.4%, 95%-CI: 19.4% to 75.6%), and seven studies reported 90-day mortality (46.4%, 95%-CI: 21.6% to 58.9%). Two studies reported 180-day mortality, four studies reported 1-year mortality, and two studies reported 2-year mortality. ICU mortality was reported in two studies.

## 4. Discussion

Our systematic review found that nearly 40% of patients receiving ECMO for HRPE died, and that the proportion of patients who suffered from cardiac arrest either prior to or during ECMO and the pre-ECMO heart rate were significantly associated with mortality. Notably, all patients in the control group received various curative therapies. Patients who received concurrent surgical embolectomy or catheter-directed therapies had comparable outcomes with ECMO as a standalone strategy. Patients receiving ECMO and systemic thrombolysis had significantly higher mortality than those receiving concomitant catheter-directed therapies.

The mortality rate for patients with HRPE and obstructive shock approaches 50%; this reaches 95% in patients with cardiac arrest. Currently, systemic thrombolysis is recommended as a first-line therapy, followed by catheter-directed thrombolysis or surgical embolectomy [5]. ECMO as a supportive therapy restores circulation and oxygenation, resolves acute right ventricular failure and obstructive shock, and improves cardiac output and end-organ perfusion [5]. It also reverses no-flow or low-flow states in cardiac arrest or profound cardiogenic shock, which, alongside metabolic derangements, might impede the return of spontaneous circulation [64,65].

ECMO as a standalone therapy with anticoagulation to treat HRPE is controversial. Some studies suggest that ECMO should be used only as an adjunct, while others have used ECMO as a bridge to recovery [45,66]. Similarly, we found that some curative therapies for patients receiving ECMO significantly reduced mortality, which suggests that ECMO might be appropriate as a bridge to curative therapy for patients with HRPE. Earlier clot resolution may further improve haemodynamics and facilitate faster weaning from ECMO, potentially reducing complications associated with ECMO use and ICU stays. As such, deciding which strategy to use involves balancing the reduction in thrombus burden and the risk of haemorrhagic complications.

Haemorrhagic complications are more commonly reported amongst those who receive systemic thrombolysis and ECMO, which might explain the higher mortality in those who received ECMO and systemic thrombolysis in our meta-analysis [67]. Notably, some ECMO centres prohibit systemic thrombolysis due to the increased risk of lethal haemorrhage [4,10]. Nearly 30% of patients receiving ECMO suffer from vascular complications, and most commonly, patients suffer from bleeding, limb ischemia, and cannula site bleeding [11]. In this review, the pooled rate of haemorrhagic complications was 35.9%, although this is likely to be an overestimation, considering those patients with multiple haemorrhagic complications. Relatedly, the higher mortality in patients treated with both ECMO and systemic thrombolysis may reflect a selection bias towards higher mortality in patients for whom thrombolysis failed.

Another interesting finding from this analysis is the lower mortality in patients treated with ECMO and catheter-directed therapies, which may be due to their less invasive and targeted effect [55]. The European Society of Cardiology recommends that ECMO may be considered in combination with surgical embolectomy or catheter-directed treatment in refractory circulatory collapse or cardiac arrest (Level IIb evidence) [5]. In 2023, The American Heart Association released a scientific statement [68] reviewing the use of ECMO in high-risk pulmonary embolism and acknowledged that it is difficult to assess the certainty of evidence surrounding ECMO as a mechanical circulatory support device unless it is used early (rather than as a salvage therapy). Our meta-analysis showed that more than half of the patients with HRPE needing ECMO survived, while patients receiving concomitant systemic thrombolysis had the highest mortality. Outcomes in patients with spontaneous recovery while on ECMO support alone were comparable to those in patients requiring ECMO and surgical embolectomy or catheter-directed therapies, while the latter group had significantly lower mortality than those who received concomitant systemic thrombolysis. Nevertheless, as evidence for ECMO in patients with PE evolves, initiating ECMO prior to cardiac arrest might be considered in HRPE.

Our study has two main strengths. First, this is a large cohort of more than 6000 patients receiving ECMO with HRPE. In addition, our review defines clear a priori analyses of secondary outcomes to minimise introducing bias through post hoc analyses [69]. As compared to the previous meta-analyses [15], we included 22 more studies (6082 patients) published after December 2020, which increases the precision of the results. The inclusion of studies was also based on a robust search strategy verified by a librarian and on comprehensive inclusion criteria. We have also applied the GRADE approach to the results of our meta-analysis, which allows for a better and more holistic clinical translation of our results. Second, we explore the novel finding that ECMO, when used concurrently with some curative therapies, demonstrates a significant survival benefit. We contacted authors for missing data wherever needed.

Nonetheless, our analysis also has several limitations. First, our study was limited by retrospective observational data. Without well-powered risk or propensity-score adjustment methods, this introduces heterogeneity with the potential to confound our analysis. In addition, given the logistical and ethical concerns of randomising patients to receive ECMO, it is difficult to ascertain if ECMO provides a survival benefit. The indications for ECMO vary between institutions; this in turn affects outcomes. The reporting of adjunctive and curative therapies was also variable, and this may be related to practices or access to techniques and devices across centres. This was further compounded by the significant publication bias for our primary outcome as evidenced by a marked increase in mortality from 42.8% to 55.2% on trim-and-fill analysis. Additionally, there are likely inherent causes of selection bias. For example, clinicians may prefer to utilise ECMO with systemic thrombolysis over surgical embolectomy in patients with rapidly progressing shock. In the presence of significant time and resources, clinicians may be likely to pursue ECMO with surgical embolectomy instead of systemic thrombolysis. Nonetheless, we accounted for some of this heterogeneity using subgroup and meta-regression analyses. In addition, sensitivity analyses suggest no substantial changes in the pooled estimate after excluding studies with high risks of bias. Second, the studies did not report on the long-term outcomes (survival, functional outcome, quality of life), which are important aspects of care. Thirdly, a multi-level meta-analysis was not feasible, as we did not have access to patient-level data. Finally, given the heterogeneous reporting of curative therapies and their timing in the context of ECMO and the limitation of study-level data, it is difficult to ascertain when and how ECMO should be used when instituted as a bridge to therapy. This is further compounded by changes in the clinical management of HRPE over time between the earliest and most recent studies in our meta-analysis (1991–2022). Undoubtedly, ECMO for HRPE is a complex domain involving multiple factors including the severity at presentation, therapies considered, the type of ECMO used, and the temporal relationship between the initiation of ECMO and curative therapies. Such granular data are often not reported. However, when they are reported, the high variability that characterises ECMO and curative therapy makes comparisons difficult. The inclusion of clear and standardised details in future studies may benefit our understanding of which subpopulations and therapies are most effective.

More than 50% of patients receiving ECMO for HRPE survive. Future studies should compare various curative therapies, with a particular focus on catheter-directed therapies with ECMO for HRPE, investigate the optimal timing of ECMO in HRPE, and elucidate longer-term outcomes for patients undergoing these treatments. While outcomes may vary based on the curative therapy used, early ECMO should be considered as a stabilising measure when treating patients with HRPE. Patients treated concurrently with systemic thrombolysis have higher mortality than those receiving ECMO alone or with other curative therapies, particularly catheter-directed therapies.

## Data Availability

This manuscript makes use of publicly available data from the included studies and their supplementary information files; therefore, no original data are available for sharing.

## Supplementary Materials

The following supporting information can be downloaded at https://www.mdpi.com/article/10.3390/jcm13010064/s1.

## Abbreviations

CI	Confidence intervals
ECMO	Extracorporeal membrane oxygenation
GRADE	Grading of Recommendations, Assessment, Development and Evaluations
HRPE	High-risk pulmonary embolism
ICU	Intensive care unit
JBI	Joanna Briggs Institute
PE	Pulmonary embolism
PROSPERO	International prospective register of systematic reviews
RR	Risk ratio
VA	Venoarterial
VV	Venovenous
