# Supplementary material for: Extracorporeal Membrane Oxygenation for Pulmonary Embolism: A Systematic Review and Meta-Analysis
Source: J Clin Med. 2023 Dec 22;13(1):64. doi: 10.3390/jcm13010064 (PMC10779708; doi:10.3390/jcm13010064)
Supplement: Supplementary file 1 [file jcm-13-00064-s001.zip › jcm-2717226-supplementary.pdf]

Extracorporeal Membrane Oxygenation for Pulmonary Embolism: A Systematic Review and Meta-Analysis

Supplementary appendix

Jonathan Jia En Boey,
Ujwal Dhundi,
Ryan Ruiyang Ling,
John Keong Chiew,
Nicole Chui-Jiet Fong,
Ying Chen,
Lukas Hobohm,
Priya Nair,
Roberto Lorusso,
Graeme MacLaren,
Kollengode Ramanathan.

Corresponding author
Kollengode Ramanathan,
Cardiothoracic Intensive Care Unit, National University Heart Centre, National University Hospital,
Level 9, 1E Kent Ridge Road, Singapore 119228
Email: surrkr@nus.edu.sg

Table of Contents

Figure S1. Funnel plot and trim-and-fill analysis of mortality for patients receiving extracorporeal membrane oxygenation for high-risk pulmonary embolism 3

Figure S2. Pooled risk ratio comparing patients receiving ECMO vs no ECMO for high-risk pulmonary embolism without Hobohm 2022 4

Figure S3. Subgroup analysis of mortality for patients receiving extracorporeal membrane oxygenation for high-risk pulmonary embolism, stratified by region 5

Figure S4. Pooled mortality amongst patients receiving extracorporeal membrane oxygenation for high-risk pulmonary embolism stratified by study type. 6

Figure S5. Pooled duration of ECMO support amongst patients receiving extracorporeal membrane oxygenation for high-risk pulmonary embolism 7

Figure S6. Pooled intensive care unit length of stay amongst patients receiving extracorporeal membrane oxygenation for high-risk pulmonary embolism 7

Figure S7. Pooled hospital length of stay amongst patients receiving extracorporeal membrane oxygenation for high-risk pulmonary embolism........ 8

Figure S8. Pooled duration of mechanical ventilation amongst patients receiving extracorporeal membrane oxygenation for high-risk pulmonary embolism 8

Figure S9. Pooled incidence of haemorrhagic complications amongst patients receiving extracorporeal membrane oxygenation for high-risk pulmonary embolism 9

Table S1. Preferred Reporting Items for Systematic reviews and Meta-Analyses (PRISMA) 2020 Checklist 10

Table S2. Search strategy for databases........ 13

Table S3. Data collection template 14

Table S4a. Demographics and outcomes of included studies 16

Table S4b. Risk factors 19

Table S4c. Definitive therapies for haemodynamically unstable pulmonary embolism........ 21

Table S4d. Details of pulmonary embolism 22

Table S4e. Patient characteristics at admission for patients with ECMO........ 24

Table S4f. Patient characteristics at admission for patients without ECMO 26

Table S4g. ECMO Characteristics........ 27

Table S4h. Subgroup demographics by region........ 29

Table S5. Joanna Briggs Institute Checklist 30

Table S6. Grading of Recommendations, Assessment, Development, and Evaluations 32

Table S7. Summary of subgroup and meta-regression analysis........ 34

Table S8: Summary of secondary outcomes........ 36

Table S9: Complications reported by included studies 37

Figure S1. Funnel plot and trim–and–fill analysis of mortality for patients receiving extracorporeal membrane oxygenation for high-risk pulmonary embolism [9,10,26-62]

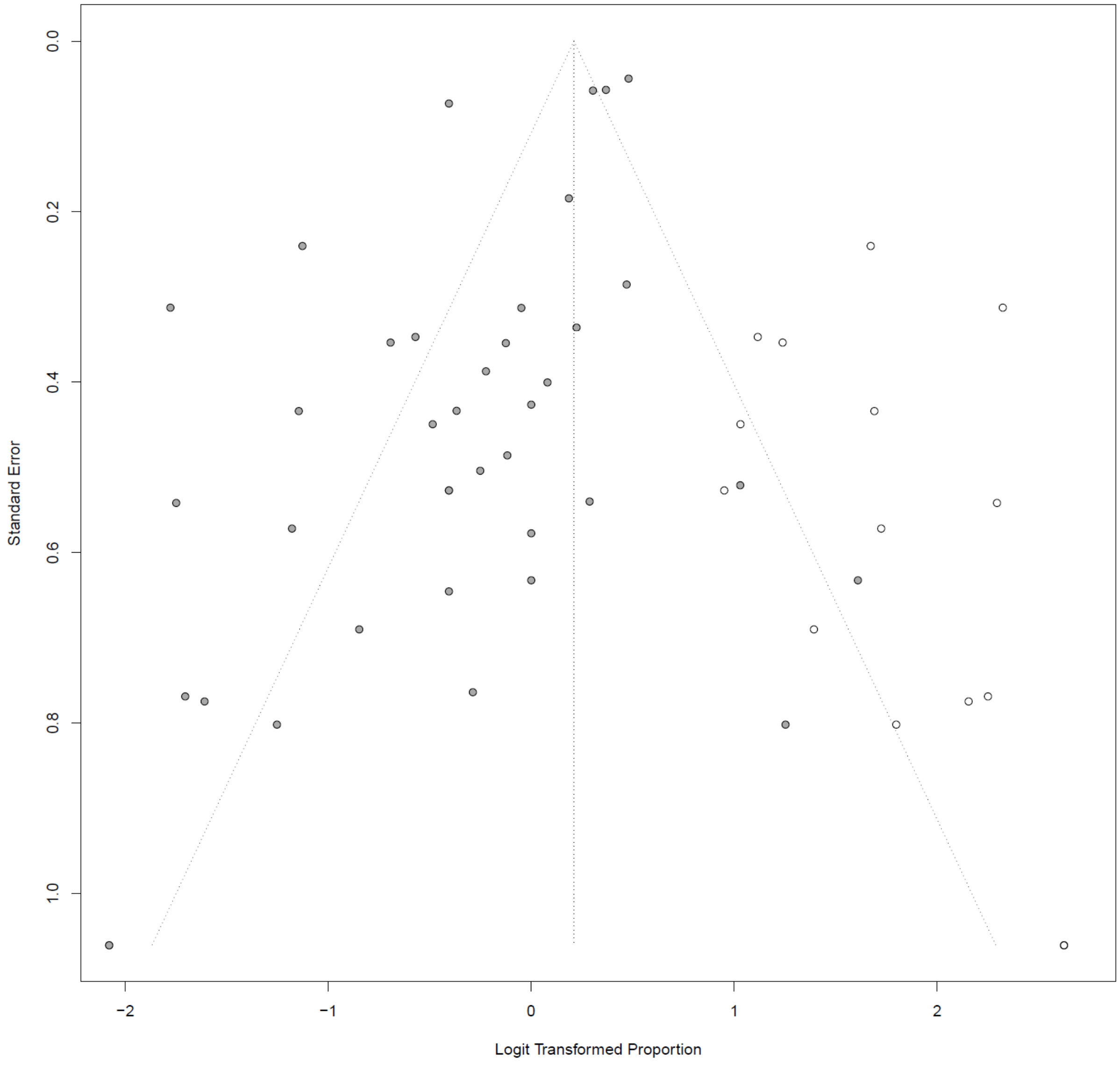

Figure S2. Pooled risk ratio comparing patients receiving ECMO vs no ECMO for high-risk pulmonary embolism without Hobohm 2022 [29,31,37,38,40,41,46,58,60,62]

Study	ECMO Nonsurvivors	ECMO Total	Control Nonsurvivors	Control Total	RR	95%-CI	Weight
Meneveau 2018	32	52	55	128	1.43	[1.07; 1.92]	17.1%
Minakawa 2018	23	94	50	261	1.28	[0.83; 1.97]	12.1%
Kjaergaard 2019	11	22	1	14	7.00	[1.01; 48.44]	1.1%
Mandigers 2019	14	19	19	20	0.78	[0.58; 1.03]	17.4%
Goldberg 2021	4	27	2	32	2.37	[0.47; 11.96]	1.5%
Hobohm 2022	1,357	2,197	183,120	1,170,157	3.95	[3.82; 4.08]	0.0%
Panholzer 2022	6	15	17	88	2.07	[0.98; 4.40]	5.8%
Tsai 2022	13	25	9	15	0.87	[0.50; 1.52]	8.9%
Nasser 2023	312	780	260	780	1.20	[1.05; 1.37]	23.8%
Kelty 2023	9	22	21	70	1.36	[0.74; 2.53]	7.8%
Sim 2023	6	15	7	24	1.37	[0.57; 3.30]	4.5%
**Random effects model**	**1,787**	**3,268**	**183,561**	**1,171,589**	**1.21**	**[0.99; 1.49]**	**100.0%**
**Prediction interval**						**[0.71; 2.06]**	

Heterogeneity: I^2 = 49%, τ^2 = 0.0417, p = 0.04

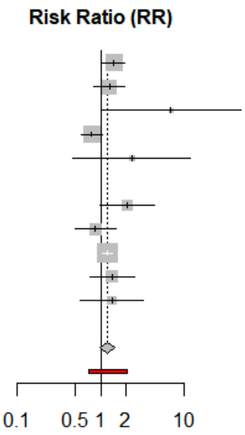

Figure S3. Subgroup analysis of mortality for patients receiving extracorporeal membrane oxygenation for high-risk pulmonary embolism, stratified by region [9,10,26-62]

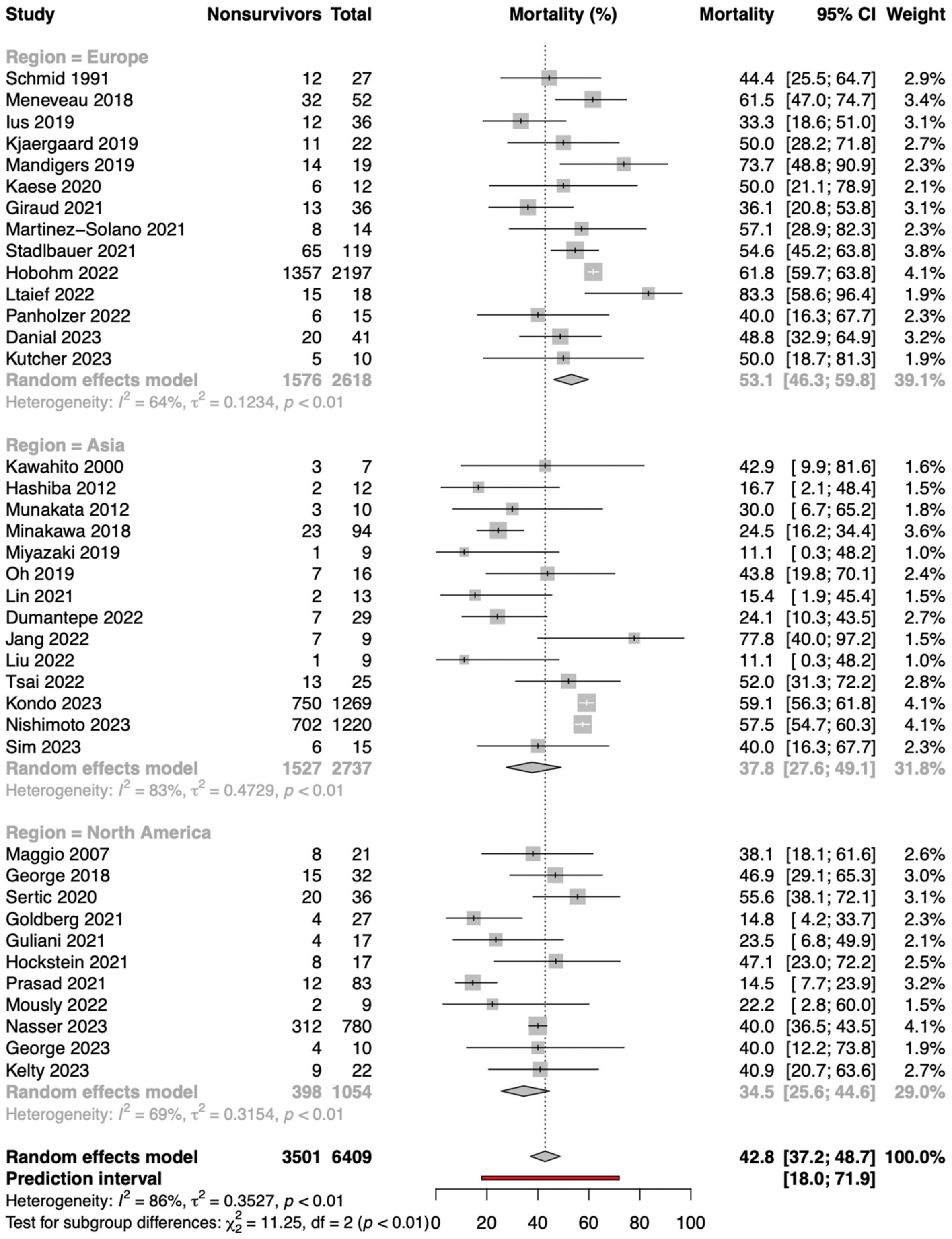

Figure S4. Pooled mortality amongst patients receiving extracorporeal membrane oxygenation for high-risk pulmonary embolism stratified by study type [9,10,26-62]

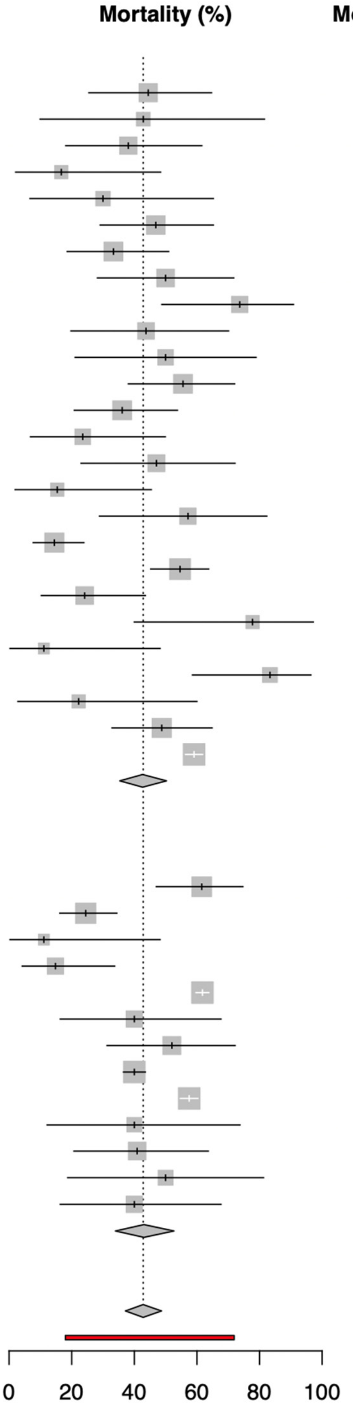

Study	Nonsurvivors	Total	Mortality	95% CI	Weight
**Type = Case Series**					
Schmid 1991	12	27	44.4	[25.5; 64.7]	2.9%
Kawahito 2000	3	7	42.9	[9.9; 81.6]	1.6%
Maggio 2007	8	21	38.1	[18.1; 61.6]	2.6%
Hashiba 2012	2	12	16.7	[2.1; 48.4]	1.5%
Munakata 2012	3	10	30.0	[6.7; 65.2]	1.8%
George 2018	15	32	46.9	[29.1; 65.3]	3.0%
Ius 2019	12	36	33.3	[18.6; 51.0]	3.1%
Kjaergaard 2019	11	22	50.0	[28.2; 71.8]	2.7%
Mandigers 2019	14	19	73.7	[48.8; 90.9]	2.3%
Oh 2019	7	16	43.8	[19.8; 70.1]	2.4%
Kaese 2020	6	12	50.0	[21.1; 78.9]	2.1%
Sertic 2020	20	36	55.6	[38.1; 72.1]	3.1%
Giraud 2021	13	36	36.1	[20.8; 53.8]	3.1%
Guliani 2021	4	17	23.5	[6.8; 49.9]	2.1%
Hockstein 2021	8	17	47.1	[23.0; 72.2]	2.5%
Lin 2021	2	13	15.4	[1.9; 45.4]	1.5%
Martinez–Solano 2021	8	14	57.1	[28.9; 82.3]	2.3%
Prasad 2021	12	83	14.5	[7.7; 23.9]	3.2%
Stadlbauer 2021	65	119	54.6	[45.2; 63.8]	3.8%
Dumantepe 2022	7	29	24.1	[10.3; 43.5]	2.7%
Jang 2022	7	9	77.8	[40.0; 97.2]	1.5%
Liu 2022	1	9	11.1	[0.3; 48.2]	1.0%
Ltaief 2022	15	18	83.3	[58.6; 96.4]	1.9%
Mously 2022	2	9	22.2	[2.8; 60.0]	1.5%
Danial 2023	20	41	48.8	[32.9; 64.9]	3.2%
Kondo 2023	750	1269	59.1	[56.3; 61.8]	4.1%
**Random effects model**	**1027**	**1933**	**42.7**	**[35.4; 50.3]**	**63.6%**
Heterogeneity: $I^2 = 78\%$, $\tau^2 = 0.3929$, $p < 0.01$					
**Type = Cohort Study**					
Meneveau 2018	32	52	61.5	[47.0; 74.7]	3.4%
Minakawa 2018	23	94	24.5	[16.2; 34.4]	3.6%
Miyazaki 2019	1	9	11.1	[0.3; 48.2]	1.0%
Goldberg 2021	4	27	14.8	[4.2; 33.7]	2.3%
Hobohm 2022	1357	2197	61.8	[59.7; 63.8]	4.1%
Panholzer 2022	6	15	40.0	[16.3; 67.7]	2.3%
Tsai 2022	13	25	52.0	[31.3; 72.2]	2.8%
Nasser 2023	312	780	40.0	[36.5; 43.5]	4.1%
Nishimoto 2023	702	1220	57.5	[54.7; 60.3]	4.1%
George 2023	4	10	40.0	[12.2; 73.8]	1.9%
Kelty 2023	9	22	40.9	[20.7; 63.6]	2.7%
Kutcher 2023	5	10	50.0	[18.7; 81.3]	1.9%
Sim 2023	6	15	40.0	[16.3; 67.7]	2.3%
**Random effects model**	**2474**	**4476**	**43.1**	**[34.0; 52.7]**	**36.4%**
Heterogeneity: $I^2 = 93\%$, $\tau^2 = 0.3376$, $p < 0.01$					
**Random effects model**	**3501**	**6409**	**42.8**	**[37.2; 48.7]**	**100.0%**
**Prediction interval**				**[18.0; 71.9]**	
Heterogeneity: $I^2 = 86\%$, $\tau^2 = 0.3527$, $p < 0.01$					
Test for subgroup differences: $\chi^2_1 = 0.00$, df = 1 ($p = 0.95$)					

Figure S5. Pooled duration of ECMO support amongst patients receiving extracorporeal membrane oxygenation for high-risk pulmonary embolism [9,10,26,28,30,33-37,40,42-45,47,49,50,52-57,59,61,62]

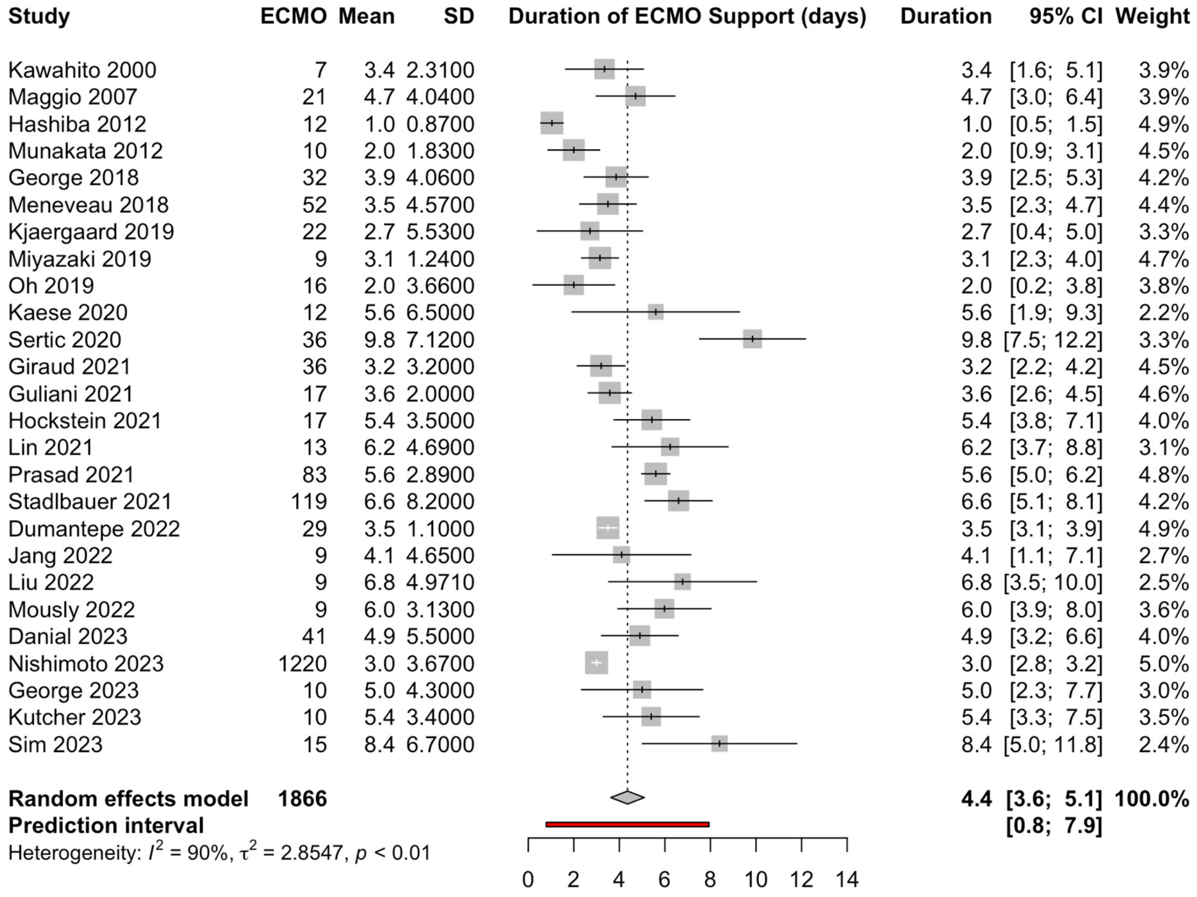

Figure S6. Pooled intensive care unit length of stay amongst patients receiving extracorporeal membrane oxygenation for high-risk pulmonary embolism [9,34,36,39,42,44-50,52,53,55,56,59,60,62]

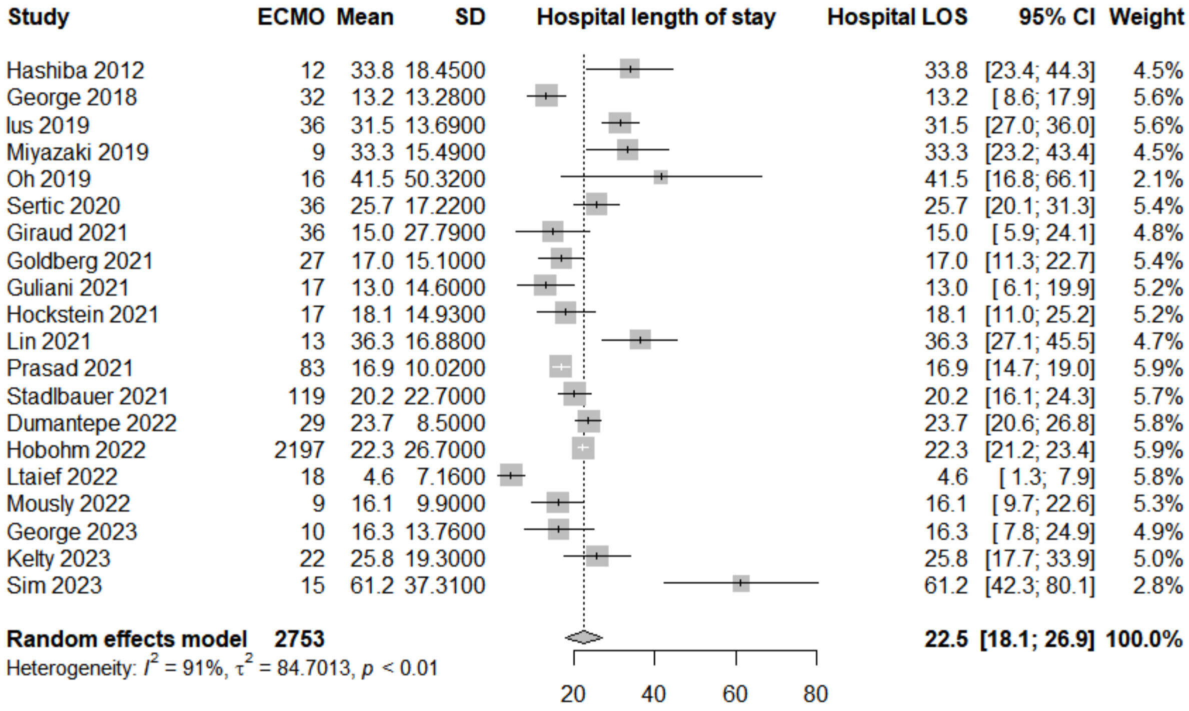

Figure S7. Pooled hospital length of stay amongst patients receiving extracorporeal membrane oxygenation for high-risk pulmonary embolism [9,34,36,39,42,44,-50,52,53,55,56,59,60,62]

Study	ECMO	Mean	SD	Hospital LOS	95% CI	Weight
Hashiba 2012	12	33.8	18.4500	33.8	[23.4; 44.3]	4.5%
George 2018	32	13.2	13.2800	13.2	[8.6; 17.9]	5.6%
Ius 2019	36	31.5	13.6900	31.5	[27.0; 36.0]	5.6%
Miyazaki 2019	9	33.3	15.4900	33.3	[23.2; 43.4]	4.5%
Oh 2019	16	41.5	50.3200	41.5	[16.8; 66.1]	2.1%
Sertic 2020	36	25.7	17.2200	25.7	[20.1; 31.3]	5.4%
Giraud 2021	36	15.0	27.7900	15.0	[5.9; 24.1]	4.8%
Goldberg 2021	27	17.0	15.1000	17.0	[11.3; 22.7]	5.4%
Guliani 2021	17	13.0	14.6000	13.0	[6.1; 19.9]	5.2%
Hockstein 2021	17	18.1	14.9300	18.1	[11.0; 25.2]	5.2%
Lin 2021	13	36.3	16.8800	36.3	[27.1; 45.5]	4.7%
Prasad 2021	83	16.9	10.0200	16.9	[14.7; 19.0]	5.9%
Stadlbauer 2021	119	20.2	22.7000	20.2	[16.1; 24.3]	5.7%
Dumantepe 2022	29	23.7	8.5000	23.7	[20.6; 26.8]	5.8%
Hobohm 2022	2197	22.3	26.7000	22.3	[21.2; 23.4]	5.9%
Ltaief 2022	18	4.6	7.1600	4.6	[1.3; 7.9]	5.8%
Mously 2022	9	16.1	9.9000	16.1	[9.7; 22.6]	5.3%
George 2023	10	16.3	13.7600	16.3	[7.8; 24.9]	4.9%
Kelty 2023	22	25.8	19.3000	25.8	[17.7; 33.9]	5.0%
Sim 2023	15	61.2	37.3100	61.2	[42.3; 80.1]	2.8%
**Random effects model**	**2753**			**22.5**	**[18.1; 26.9]**	**100.0%**
**Prediction interval**					**[2.6; 42.4]**	

Heterogeneity: $I^2 = 91\%$, $\tau^2 = 84.7013$, $p < 0.01$

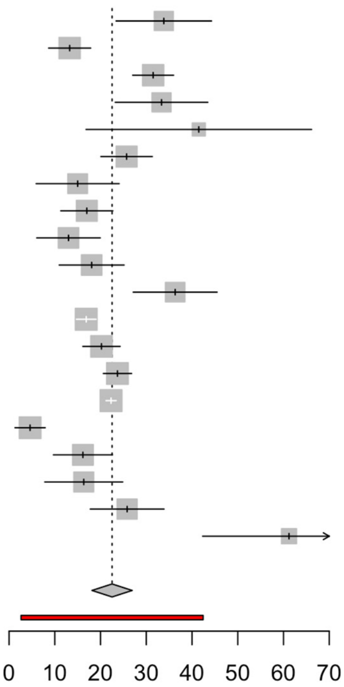

Figure S8. Pooled duration of mechanical ventilation amongst patients receiving extracorporeal membrane oxygenation for high-risk pulmonary embolism [45,49,52,55,57]

Study	ECMO	Mean	SD	Duration	95% CI	Weight
Giraud 2021	36	2.4	8.1100	2.4	[-0.2; 5.0]	20.0%
Hockstein 2021	17	10.8	7.2100	10.8	[7.4; 14.3]	19.1%
Prasad 2021	83	9.6	15.2800	9.6	[6.3; 12.9]	19.3%
Stadlbauer 2021	119	12.2	14.1500	12.2	[9.7; 14.7]	20.1%
Ltaief 2022	18	1.4	1.3700	1.4	[0.8; 2.1]	21.4%
**Random effects model**	**273**			**7.2**	**[2.7; 11.6]**	**100.0%**
**Prediction interval**					**[-10.1; 24.4]**	

Heterogeneity: $I^2 = 96\%$, $\tau^2 = 24.1222$, $p < 0.01$

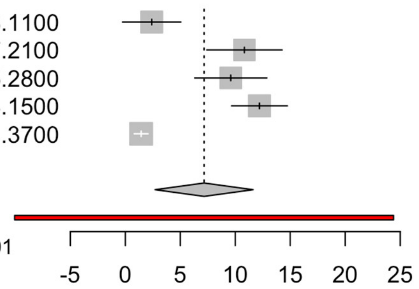

Figure S9. Pooled incidence of haemorrhagic complications amongst patients receiving extracorporeal membrane oxygenation for high-risk pulmonary embolism [9,10,26,28-31,36,37,39,41-47,49,50,52-55,58,62]

Study	Complications	Patients	Events	95%-CI	Weight
Maggio 2007	18	21	85.7	[63.7; 97.0]	3.7%
Munakata 2012	10	10	100.0	[69.2; 100.0]	1.8%
George 2018	9	32	28.1	[13.7; 46.7]	4.2%
Meneveau 2018	20	52	38.5	[25.3; 53.0]	4.5%
Ius 2019	2	36	5.6	[0.7; 18.7]	3.4%
Mandigers 2019	14	19	73.7	[48.8; 90.9]	3.9%
Miyazaki 2019	2	9	22.2	[2.8; 60.0]	3.2%
Oh 2019	9	16	56.2	[29.9; 80.2]	4.0%
Kaese 2020	4	12	33.3	[9.9; 65.1]	3.7%
Sertic 2020	14	36	38.9	[23.1; 56.5]	4.4%
Giraud 2021	18	36	50.0	[32.9; 67.1]	4.4%
Goldberg 2021	16	27	59.3	[38.8; 77.6]	4.2%
Guliani 2021	5	17	29.4	[10.3; 56.0]	3.9%
Hockstein 2021	2	17	11.8	[1.5; 36.4]	3.3%
Lin 2021	4	13	30.8	[9.1; 61.4]	3.7%
Prasad 2021	17	83	20.5	[12.4; 30.8]	4.5%
Dumantepe 2022	7	29	24.1	[10.3; 43.5]	4.1%
Jang 2022	7	9	77.8	[40.0; 97.2]	3.2%
Liu 2022	1	9	11.1	[0.3; 48.2]	2.6%
Ltaief 2022	13	18	72.2	[46.5; 90.3]	3.9%
Panholzer 2022	8	15	53.3	[26.6; 78.7]	3.9%
Tsai 2022	13	25	52.0	[31.3; 72.2]	4.2%
Danial 2023	11	41	26.8	[14.2; 42.9]	4.3%
Nasser 2023	150	780	19.2	[16.5; 22.2]	4.7%
Nishimoto 2023	39	1220	3.2	[2.3; 4.3]	4.6%
Sim 2023	3	15	20.0	[4.3; 48.1]	3.6%
**Random effects model**		**2597**	**35.9**	**[25.5; 47.8]**	**100.0%**
**Prediction interval**				**[4.7; 86.4]**	

Heterogeneity: $I^2 = 93\%$, $\tau^2 = 1.3255$, $p < 0.01$

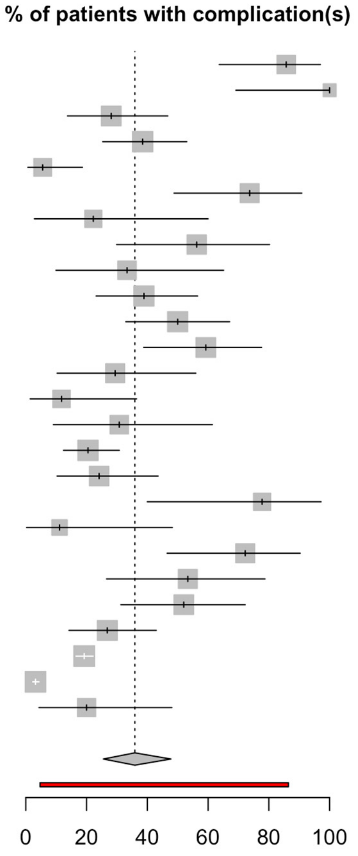

Table S1. Preferred Reporting Items for Systematic reviews and Meta-Analyses (PRISMA) 2020 Checklist

Section and Topic	Item #	Checklist item	Location where item is reported
**TITLE**			
Title	1	Identify the report as a systematic review.	Page 1
**ABSTRACT**			
Abstract	2	See the PRISMA 2020 for Abstracts checklist.	Page 3
**INTRODUCTION**			
Rationale	3	Describe the rationale for the review in the context of existing knowledge.	Page 4
Objectives	4	Provide an explicit statement of the objective(s) or question(s) the review addresses.	Page 4
**METHODS**			
Eligibility criteria	5	Specify the inclusion and exclusion criteria for the review and how studies were grouped for the syntheses.	Page 4-5
Information sources	6	Specify all databases, registers, websites, organisations, reference lists and other sources searched or consulted to identify studies. Specify the date when each source was last searched or consulted.	Page 4
Search strategy	7	Present the full search strategies for all databases, registers and websites, including any filters and limits used.	Table S2
Selection process	8	Specify the methods used to decide whether a study met the inclusion criteria of the review, including how many reviewers screened each record and each report retrieved, whether they worked independently, and if applicable, details of automation tools used in the process.	Page 5
Data collection process	9	Specify the methods used to collect data from reports, including how many reviewers collected data from each report, whether they worked independently, any processes for obtaining or confirming data from study investigators, and if applicable, details of automation tools used in the process.	Page 5
Data items	10a	List and define all outcomes for which data were sought. Specify whether all results that were compatible with each outcome domain in each study were sought (e.g. for all measures, time points, analyses), and if not, the methods used to decide which results to collect.	Table S3
	10b	List and define all other variables for which data were sought (e.g. participant and intervention characteristics, funding sources). Describe any assumptions made about any missing or unclear information.	Table S3
Study risk of bias	11	Specify the methods used to assess risk of bias in the included studies, including details of the tool(s) used, how many reviewers assessed	Page 5, Table

assessment		each study and whether they worked independently, and if applicable, details of automation tools used in the process.	S5
Effect measures	12	Specify for each outcome the effect measure(s) (e.g. risk ratio, mean difference) used in the synthesis or presentation of results.	Page 5-6
Synthesis methods	13a	Describe the processes used to decide which studies were eligible for each synthesis (e.g. tabulating the study intervention characteristics and comparing against the planned groups for each synthesis (item #5)).	Page 5-6
	13b	Describe any methods required to prepare the data for presentation or synthesis, such as handling of missing summary statistics, or data conversions.	Page 5-6
	13c	Describe any methods used to tabulate or visually display results of individual studies and syntheses.	Page 5-6, Table S4
	13d	Describe any methods used to synthesize results and provide a rationale for the choice(s). If meta-analysis was performed, describe the model(s), method(s) to identify the presence and extent of statistical heterogeneity, and software package(s) used.	Page 5-6
	13e	Describe any methods used to explore possible causes of heterogeneity among study results (e.g. subgroup analysis, meta-regression).	Page 5-6
	13f	Describe any sensitivity analyses conducted to assess robustness of the synthesized results.	Page 5-6
Reporting bias assessment	14	Describe any methods used to assess risk of bias due to missing results in a synthesis (arising from reporting biases).	Table S5
Certainty assessment	15	Describe any methods used to assess certainty (or confidence) in the body of evidence for an outcome.	Table S6
**RESULTS**			
Study selection	16a	Describe the results of the search and selection process, from the number of records identified in the search to the number of studies included in the review, ideally using a flow diagram.	Page 6-7, Figure 1
	16b	Cite studies that might appear to meet the inclusion criteria, but which were excluded, and explain why they were excluded.	Page 6-7
Study characteristics	17	Cite each included study and present its characteristics.	Page 7, Table S4
Risk of bias in studies	18	Present assessments of risk of bias for each included study.	Table S5
Results of individual studies	19	For all outcomes, present, for each study: (a) summary statistics for each group (where appropriate) and (b) an effect estimate and its precision (e.g. confidence/credible interval), ideally using structured tables or plots.	Page 6-9

Results of syntheses	20a	For each synthesis, briefly summarise the characteristics and risk of bias among contributing studies.	Table S6
	20b	Present results of all statistical syntheses conducted. If meta-analysis was done, present for each the summary estimate and its precision (e.g. confidence/credible interval) and measures of statistical heterogeneity. If comparing groups, describe the direction of the effect.	Page 6-9
	20c	Present results of all investigations of possible causes of heterogeneity among study results.	Page 6-9
	20d	Present results of all sensitivity analyses conducted to assess the robustness of the synthesized results.	Page 6-9
Reporting biases	21	Present assessments of risk of bias due to missing results (arising from reporting biases) for each synthesis assessed.	Table S6
Certainty of evidence	22	Present assessments of certainty (or confidence) in the body of evidence for each outcome assessed.	Table S6
**DISCUSSION**			
Discussion	23a	Provide a general interpretation of the results in the context of other evidence.	Page 9-12
	23b	Discuss any limitations of the evidence included in the review.	Page 9-12
	23c	Discuss any limitations of the review processes used.	Page 9-12
	23d	Discuss implications of the results for practice, policy, and future research.	Page 9-12
**OTHER INFORMATION**			
Registration and protocol	24a	Provide registration information for the review, including register name and registration number, or state that the review was not registered.	Page 4
	24b	Indicate where the review protocol can be accessed, or state that a protocol was not prepared.	Page 4
	24c	Describe and explain any amendments to information provided at registration or in the protocol.	Page 5-6
Support	25	Describe sources of financial or non-financial support for the review, and the role of the funders or sponsors in the review.	Page 5
Competing interests	26	Declare any competing interests of review authors.	Page 13
Availability of data, code and other materials	27	Report which of the following are publicly available and where they can be found: template data collection forms; data extracted from included studies; data used for all analyses; analytic code; any other materials used in the review.	Table S3

From: Page MJ, McKenzie JE, Bossuyt PM, Boutron I, Hoffmann TC, Mulrow CD, et al. The PRISMA 2020 statement: an updated guideline for reporting systematic reviews. BMJ 2021;372:n71. doi: 10.1136/bmj.n71

Table S2. Search strategy for databases

MEDLINE via PubMed

No.	Search	Results
1	(("Pulmonary Embolism"[MeSH Terms]) OR (pulmonary embol*[Title/Abstract] OR PE[title/abstract])) and (("Extracorporeal Membrane Oxygenation"[Mesh]) OR ((Extracor*[Title/Abstract] OR extra-cor*[Title/Abstract]) and ((membra*[Title/Abstract] AND oxygen*[Title/Abstract]) OR (life support[Title/Abstract] OR lung support[Title/Abstract] OR circulat*[Title/Abstract]))) OR (ecls[Title/Abstract] OR ecmo[Title/Abstract]))	870

EMBASE via Ovid

No.	Search	Results
1	((veno-arterial ECMO/ or arterio-venous ECMO/ or veno-venous ECMO/) OR (extracorporeal oxygenation/) OR (((Extracor* or extra-cor*) and (membra* oxygen* or life support or lung support or circulat*)).ti,ab) OR ((ECLS or ECMO).ab,ti)) AND lung embolism/	2475

Cochrane Library

No.	Search	Results
1	MeSH descriptor: [Extracorporeal Membrane Oxygenation] explode all trees	
2	((Extracor* OR Extra-cor*) AND ((membra* oxygen*) OR life support OR lung support OR circulat*) OR ECLS OR ECMO):ti,ab	
3	MeSH descriptor: [Pulmonary Embolism] explode all trees	
4	(Pulmonary AND Embol* OR PE):ti,ab	
5	(1 OR 2) AND (3 OR 4)	124

Scopus

No.	Search	Results
1	TITLE-ABS ((extracor* OR extra-cor*) AND ((membra* AND oxygen*) OR life AND support OR lung AND support OR circulat*) OR ecls OR ecmo) AND TITLE-ABS ((pulmonary AND embol*) OR pe)	415

* corresponds the use of a wildcard search in the above search strategies

Table S3. Data collection template

**Study Characteristics**	Authors
	Title
	Year of Publication
	Country / Hospital
	Duration of Study
	Inclusion/exclusion criteria
	Sample Size
**Patient Demographics**	Age
	Number of male patients
	Ethnicity / Race
**Number and mortality of patients receiving ECMO and any adjuvant treatment**	Bridged to surgical embolectomy with ECMO
	Bridged to catheter-directed therapy with ECMO
	Bridged to thrombolysis with ECMO
	Bridged to recovery with ECMO post-surgical embolectomy
	Bridged to recovery with ECMO after catheter-directed therapy
	Bridged to recovery with ECMO post-thrombolysis
	ECMO Only
	Others
**Number and mortality of patients of control groups and any adjuvant treatment**	Surgical embolectomy
	Catheter-directed therapy
	Thrombolysis
	Others
**Comorbidities**	>60yo
	Hypertension
	Diabetes Mellitus
	Hypersensitivity lung disease
	Obesity
	COPD/Asthma
	Coronary Artery Disease
	Smoking
	Deep venous thrombosis or history of deep venous thrombosis
	Malignancy
	Immobility
	Recent operation less than one month ago
**Pulmonary Embolism Characteristics**	Location
	Type
	History of pulmonary embolism
**Pre-ECMO characteristics**	Indication
	Organ failure
	Organ failure scores (APACHE, SOFA, SAPS, LIS)
	PaO2/FiO2 ratio
	PO2
	PCO2
	FiO2
	acute respiratory distress syndrome
	pH
	lactate
	D-dimer
	fibrinogen
	IL-6
	white blood cell count
	time from mechanical ventilation to ECMO
**ECMO characteristics**	Number of VV-ECMO
	Number of VA-ECMO
	Cannulation site
**Ventilatory parameters**	Tidal volume
	Peak inspiratory pressure
	Plateau pressure
	Driving pressure
	Mean arterial pressure

	Respiratory rate
	Positive end-expiratory pressure
**Outcomes**	Mortality
	Survivors (short - 30 days, long - >3mths)
	ICU length of stay
	Hospital length of stay
	Duration of mechanical ventilation
	Duration on ECMO
	Complications while on ECMO

Table S4a. Demographics and outcomes of included studies [9,10,26-62]

Author	Country	No. of patients	Ethnicity	Patients with ECMO								Patients without ECMO			
				Sample Size	Mortality (number)	Male (number)	Age (yrs)	Duration of ECMO (days)	Duration of MV (days)	Length of ICU stay (days)	Length of hospital stay (days)	Sample Size	Mortality	Male	Age (yrs)
Schmid 1991	Germany	27		27	12	17	50 ± 14.51								
Kawahito 2000	Japan	7	7 Japanese	7	3	2	61 ± 16	3.35 ± 2.31							
Maggio 2007	USA	21		21	8	10	40.52 ± 17.01	4.71 ± 4.04							
Hashiba 2012	Japan	12		12	2	4	65.2 ± 15.7	1.04 ± 0.87			33.83 ± 18.45				
Munakata 2012	Japan	10		10	3	2	57.75 ± 20.47	2 ± 1.83							
George 2018	USA	32		32	15	17	56.13 ± 5.89	3.86 ± 4.06		6.78 ± 7.22	13.25 ± 13.28				
Meneveau 2018	France	180		52	32	27	47.6 ± 15	3.5 ± 4.57		7.17 ± 10.9		128	55	69	64 ± 15
Minakawa 2018	Japan	355		94	23							261	50		
Ius 2019	Germany	36		36	12	23	52.25 ± 14.4			15.5 ± 6.61	31.5 ± 13.69				
Kjaergaard 2019	Denmark	36		22	11	10	55 ± 15.53	2.71 ± 5.53				14	1	7	59.29 ± 15.17
Mandigers 2019	Netherlands	39		19	14	8	43.33 ± 23.94					20	19	8	55.33 ± 14.42
Miyazaki 2019	Japan	9		9	1	4	51.5 ± 15.49	3.15 ± 1.24		8.67 ± 2.69	33.33 ± 15.49				
Oh 2019	South Korea	16		16	7	6	53.33 ± 26.82	2 ± 3.66		11.35 ± 13.34	41.48 ± 50.32				
Kaese 2020	Germany	12		12	6	9	44.2 ± 11.9	5.6 ± 6.5		22.4 ± 23					

Sertic 2020	USA	36		36	20	16	51.36 ± 14.05	9.85 ± 7.12		18.98 ± 12.56	25.7 ± 17.22				
Giraud 2021	Switzerland	36		36	13	27	57 ± 17.76	3.2 ± 3.2	2.4 ± 8.11	8.7 ± 6.18	15 ± 27.79				
Guliani 2021	New Mexico	17		17	4	9	54.11 ± 29.25	3.58 ± 2		9 ± 12.1	13 ± 14.6				
Goldberg 2021*	USA	59	ECMO first: 11 African American SE first: 12 African American	27	4	10	55.7 ± 17.7				17 ± 15.1	32	2	20	55.4 ± 12.6
Hockstein 2021	USA	17		17	8	8	54.67 ± 21.02	5.43 ± 3.5	10.83 ± 7.21	13 ± 9.82	18.06 ± 14.93				
Lin 2021	Taiwan	13		13	2	6	64 ± 11.58	6.23 ± 4.69			36.31 ± 16.88				
Martinez-Solano 2021	Spain	14		14	8										
Prasad 2021	USA	83	27 White 37 African American	83	12	51	53.33 ± 16.64	5.6 ± 2.89	9.59 ± 15.28		16.87 ± 10.02				
Stadlbauer 2021	Germany	119		119	65	69	50.9 ± 14.8	6.6 ± 8.2	12.2	16.6 ± 18.4	20.2 ± 22.7				
Dumantepe 2022	Turkey	29		29	7	17	55.3 ± 9.2	3.5 ± 1.1		9.9 ± 1.6	23.7 ± 8.5				
Hobohm 2022*	Germany	1172354		2197	1357	1358	54.67 ± 15.58				22.3 ± 26.7	1170157	183120	544174	70.67 ± 14.82
Jang 2022	South Korea	9		9	7	6	54.5 ± 18.18	4.1 ± 4.65							
Ltaief 2022	Switzerland	18		18	15	9	56.75 ± 5.22		1.44	3.57 ± 6.03	4.6 ± 7.16				
Mously 2022	USA	9		9	2	2	52.5 ± 13.47	5.98 ± 3.13		11.75 ± 8.08	16.15 ± 9.9				

Panholzer 2022	Germany	103		15	6							88	17		
Tsai 2022	Taiwan	40		25	13	17	53 ± 18.08					15	9	5	72.67 ± 13.9
Danial 2023	France	41		41	20	21	47 ± 12.1	4.9 ± 5.5		31.6 ± 60.1	4.9 ± 5.5				
Kondo 2023	Japan	1318		1269	750										
Nasser 2023	USA	1560		780	312							780	260		
Nishimoto 2023	Japan	1220		1220	702	490	59.29 ± 16.02	3 ± 3.67		24.41 ± 48.96	3 ± 3.67				
George 2023	USA	10		10	4	6	55 ± 14.5	5 ± 4.3			16.33 ± 13.76				
Kelty 2023	USA	92	White 19	22	9	9	53.45 ± 14.57				25.81 ± 19.3	70	21	42	58.41 ± 16.85
Kutcher 2023	Switzerland	15		10	5			5.4 ± 3.4							
Sim 2023	Korea	39		15	6			8.4 ± 6.7			61.2 ± 37.31	24	7		

Abbreviations: ECMO: extracorporeal membrane oxygenation, ICU: intensive care unit, MV: mechanical ventilation, SE: surgical embolectomy, USA: United States of America

Data presented as mean ± standard deviation.
*Values derived from combining subgroup data provided by the studies

Table S4b. Risk factors [9,10,26-62]

Author	Patients with ECMO	Patients without ECMO	Patients with ECMO													Patients without ECMO												
			>60 yo	HTN	DM	HLD	Obesity	COPD / Asthma	CAD	Smoking	Thrombosis	Malignancy	Immobility	Recent operation	Other	>60 yo	HTN	DM	HLD	Obesity	COPD / Asthma	CAD	Smoking	Thrombosis	Malignancy	Immobility	Recent operation	Other
Schmid 1991	27												13															
Kawahito 2000	7										2	1	1	1	1													
Maggio 2007	21		2								11		13	6	6													
Hashiba 2012	12		Not reported																									
Munakata 2012	10								5					3														
George 2018	32			14	12				4		3	4																
Meneveau 2018	52	128		14				1	3	12	20	4	25	10	11						8			7	34			12
Minakawa 2018	94	261																										
Ius 2019	36		Not reported																									
Kjaergaard 2019	22	14	18									3		16														
Mandigers 2019	19	20						1	0												5	4						
Miyazaki 2019	9		2								1		3	1														
Oh 2019	16			4	4					2	1	6	3	5	5													
Kaese 2020	12											1																
Sertic 2020	36			21	11						15	7		17														
Giraud 2021	36			5										18	3													
Guliani 2021	17										2		1	5	1													
Goldberg 2021	27	32			6			7	2	6	8	4		9				11			10	2	7	2	7		10	
Hockstein 2021	17			10	4					3	3	2	4	4	15													
Lin 2021	13											5	6	6	1													
Martinez-Solano 2021	14		Not reported																									
Prasad 2021	83								12	31	13	10	12	16														
Stadlbauer 2021	119		Not reported																									
Dumantepe 2022	29			14				5		16	9	5	11	3	11													
Hobohm 2022	2197	1170157		680	429		319	198	429			256			13		507979	160700		111804	120284	160700			236003			13141
Jang 2022	18				2					3	1			2	1													
Ltaief 2022	9		9	7	2			1			10	3		8	6													
Mously 2022	25			4	3	2				3	2	1		5	1													
Panholzer 2022	103	88	Not reported																									
Tsai 2022	27	15		8	3	3			4		6	7	6	15	2		6	4	2			3		1	3	7	1	2 CHF

																												3 CKD<br>5 chronic lung disease<br>2 CVA
Danial 2023	41			11	4	4			2	12			1		2													
Kondo 2023	1318	Not reported																										
Nasser 2023	1560	780	Not reported																									
Nishimoto 2023	1220			148	35			19	70			56			87													
George 2023	10	Not reported																										
Kelty 2023	92	70			3			5							29			15			4							88
Kutcher 2023	15	Not reported																										
Sim 2023	39	24	Not reported																									

Abbreviations: CAD: Coronary artery disease, COPD: Chronic obstructive pulmonary disease, DM: Diabetes Mellitus, ECMO: Extracorporeal membrane oxygenation, HLD: Hyperlipidaemia, HTN: Hypertension

Table S4c. Definitive therapies for haemodynamically unstable pulmonary embolism [9,10,26-62]

Author	Patients with ECMO	Patients without ECMO	Patients with ECMO										Patients without ECMO					
			With definitive treatment		ECMO Only		ECMO + SE		ECMO + CDT		ECMO + ST		SE		CDT		ST	
			Total	Deaths	Total	Deaths	Total	Deaths	Total	Deaths	Total	Deaths	Total	Deaths	Total	Deaths	Total	Deaths
Schmid 1991	27		27	12			27	12										
Kawahito 2000	7		7	3			3	1			4	2						
Maggio 2007	21		13	8	8	0	11	1										
Munakata 2012	10		10	3					8	2	2	1						
George 2018	32		22	11			2	2	15	4	5	5						
Meneveau 2018	52	128	34	18	18	14	17	7			17	13	8	1			68	24
Ius 2019	36		20	1	16	11	20	1										
Mandigers 2019	19	20	18		1						18						12	
Miyazaki 2019	9		5	0	4	1			1	0	4	0						
Oh 2019	16		13	6	3	1	9	4			7	3						
Sertic 2020	36		16	7	20	13	3	2	6	2	7	3						
Giraud 2021	36		17	11	19	2			5	1	16	10						
Guliani 2021	17		10		3				10									
Goldberg 2021	27	32	10	1	21	4	6	1			4	0	32	2				
Hockstein 2021	17				5	1	1	0	3	2	8	5						
Lin 2021	13		13	2					4	1	9	1						
Prasad 2021	83		42		41		16		11		18	2						
Stadlbauer 2021	119		69		52		17				50							
Dumantepe 2022	29		29	7					29	7								
*Hobohm 2022	2197	1170157	526	365	629	385	304	160			222	162	1478				48759	
Jang 2022	18		3	3	6	0	1	1	1	1	2	2						
Liu	9		5	0	4	1			5	0								
Ltaief 2022	9		10	6	8	7	4	3	1	0	9	6						
Mously 2022	25		9	2					9	2								
Panholzer 2022	15	88																
Tsai 2022	27	15	9	2	16	11	0	0	6	0	3	2	0	0	2	1	1	1
Nishimoto 2023	1220		432	255	788	477					432	225						
Kutcher 2023	10		10	5					10	5								
Sim 2023	15	24	15	6							15	6						

* Values derived by combining subgroup data provided by studies
Abbreviations: CDT: catheter-directed therapies, ECMO: extracorporeal membrane oxygenation, SE: surgical embolectomy, ST: systemic thrombolysis
Studies not reporting on definitive therapies are excluded from this table

Table S4d. Details of pulmonary embolism [9,10,26-62]

Author	Patients with ECMO	Patients without ECMO	Patients with ECMO				
			PE Location	PE Type	Past Hx of PE	CTPA Results	TTE Results
Schmid 1991	27					8 positive	1 positive
Kawahito 2000	7			Fulminant / massive		7 positive	
Maggio 2007	21		nil	Massive: causing sufficient obstruction to pulmonary flow to result in hemodynamic instability, right ventricular failure, and hypoxemia	2	14 positive	15 RV failure<br>2 minimal RV dysfunction
Munakata 2012	10		10 proximal lobular arteries<br>- 6 including main pulmonary artery				
Meneveau 2018	52	128	42 proximal				
Ius 2019	36		central pulmonary vessels 36,<br>Right heart chanmber 8	thrombus			
Oh 2019	16					12 out of 13 who underwent CTPA showed RV strain	Right ventricular dilatation and dysfunction
Kaese 2020	12		1 right atrium into RV			7 patients confirmed diagnosis by CT	
Sertic 2020	36				10		
Prasad 2021	83		Saddle: 37<br>Lobar: 41			79 diagnosed by CTPA	
Dumantepe 2022	29		11 saddle PE<br>6 clot-in-transit in right atrium<br>29 proximal lobar or lobular arteries<br>11 massive thrombus in main pulmonary arteries				
Jang 2022	18		8 bilateral pulmonary embolism			8 thrombus visualised<br>7 RV strain on CTPA	
Ltaief 2022	9					13 positive	
Mously 2022	25					8	
George 2023	10		Saddle pulmonary embolus: 7<br>Extensive bilateral pulmonary emboli: 3<br>Clot in transit: 2			Moderate to severe RV strain : 10	
Kelty 2023	22	70					
Kutcher 2023	10				Cardiac arrest: 4<br>Obstructive shock: 8	All confirmed diagnosis by CTPA	

					Persistent arterial hypotension: 3<br>At least one contraindication to thrombolysis: 15			
Sim 2023	15	24	Large bilateral thromboembolism: 39					

Studies not reporting relevant details have been removed from this table. Details are included only for ECMO population.

Abbreviations: CT: computed tomography, CTPA: computed tomography pulmonary angiogram, LVEF: left ventricular ejection fraction, PAP: pulmonary artery pressure, PE: pulmonary embolism, RV: right ventricle, RVSP: right ventricular systolic pressure

Table S4e. Patient characteristics at admission for patients with ECMO [9,10,26-62]

Author	Number of patients	ECPR	Cardiac arrest	Systolic BP	MAP	Heart Rate	Troponin I	RV Abnormality	Inotropic or vasopressor Use	pH	Lactate	SAPS II	SAPS III	APACHE
Schmid 1991	27		16						11					
Kawahito 2000	7		3					7						
Maggio 2007	21	10	8					17	12	7.13 ± 0.18				
Hashiba 2012	12		5	66.33 ± 21.13		121 ± 22				7.01 ± 0.25				
Munakata 2012	10		9					10	10					
George 2018	32	15	15						32		7.51 ± 6.48			
Meneveau 2018	52		39	91.9 ± 47.6		94 ± 51	2.58 ± 3.16	53	49	7.18 ± 0.24	7.36 ± 6.8			
Ius 2019	36		15						36					
Kjaergaard 2019	22		17											
Mandigers 2019	19		19							6.78 ± 0.15	14 ± 4.25			
Miyazaki 2019	9	19	9					9		6.98 ± 0.51	14.67 ± 12.25			
Oh 2019	16	12	12					15						
Kaese 2020	12	3	11					5						
Sertic 2020	36	12	22					36	36					
Giraud 2021	36	13	22	76 ± 41.69	59 ± 27.02	108 ± 36.29		36		7.08 ± 0.29	8.3 ± 8.57			
Guliani 2021	17	10	10	88.47 ± 41.83		111.91 ± 57.21				7.1 ± 0.38	10.97 ± 13.48			
Goldberg 2021	27		10	81 ± 19		112 ± 25								

Hockstein 2021	17					117.84 ± 29.85	1.57 ± 3.2			7.07 ± 0.29	9.64 ± 7.19			
Lin 2021	13		13											
Prasad 2021	83	15	23				1.85 ± 5.64	52			4.74 ± 3.55			
Stadlbauer 2021	119				55 ± 17					7.2 ± 0.17	9.6 ± 6.9			
Dumantepe 2022	29			76.4 ± 8.9	55.6 ± 7.7		2.12 ± 0.37			7.11 ± 0.13	12.5 ± 2.6			
Hobohm 2022	2197		992					1209						
Jang 2022	9		9		56.75 ± 40.07	80.75 ± 41.41								
Ltaief 2022	18		16		76.67 ± 17.7	95 ± 32.98				7.08 ± 0.34	13.73 ± 8.93			
Mously 2022	9		6				1.22 ± 0.53				8.33 ± 4.55			
Panholzer 2022	15		9											
Tsai 2022	25		15				0.15 ± 0.24			7.13 ± 0.24				
Danial 2023	41									7.1 ± 0.23	10.5 ± 7.8	70.1 ± 19.4		
Nishimoto 2023	1220		736											
George 2023	10	6												
Kelty 2023	22			101.32 ± 31.73	77.23 ± 19.56			RV dilated: 9<br>RV function reduced: 14<br>Septal flattening: 7		7.2 ± 0.2	4.54 ± 3.7			
Kucher 2023	10									7.22 ± 0.19				
Sim 2023	15							RV ventricular dilatation and flattening of interventricular septum: 15						

Studies not reporting relevant details have been removed from this table.

Abbreviations: APACHE: Acute Physiology and Chronic Health Evaluation, BP: blood pressure, ECPR: extracorporeal cardiopulmonary resuscitation, MAP: mean arterial pressure, RV: right ventricle, SAPS: Simplified Acute Physiology Score

Table S4f. Patient characteristics at admission for patients without ECMO [9,10,26-62]

Author	Number of patients	Patients with cardiac arrest	Systolic BP	MAP	Heart Rate	Troponin I	RV Abnormality	Inotropic or vasopressor Use	pH	Lactate	SOFA Score	SAPS II	SAPS III	APACHE
Meneveau 2018	128	45	98.6 ± 29.7		102 ± 22	8.95 ± 19.34		111						
Moon 2018	9	8					7		7.2 ± 0.3					
Mandigers 2019	20								6.51 ± 0.97	14.5 ± 6.86				
Goldberg 2021	32	12	90 ± 24		113 ± 14									
Hobohm 2022	1170157	76204					332292							
Panholzer 2022	99	9												
Tsai 2022	15	3				0.43 ± 0.7			7.26 ± 0.13					
Nasser 2023	780													
Kelty 2023	70		101.32 ± 31.73	77.23 ± 19.56					7.22 ± 0.19	4.54 ± 3.7	9.55 ± 3.14			

Studies not reporting relevant details have been removed from this table.

Abbreviations: APACHE: Acute Physiology and Chronic Health Evaluation, BP: blood pressure, MAP: mean arterial pressure, RV: right ventricle, SAPS: Simplified Acute Physiology Score

Table S4g. ECMO Characteristics [9,10,26-62]

Author	Number of patients	ECMO Cannula Site	VA-ECMO	VA-ECMO Mortality	VV-ECMO	VV-ECMO Mortality	ECMO Flow Rate
Schmid 1991	27	Aortic and right atrial cannulas	27				
Kawahito 2000	7	Fem-fem (15Fr art, 21Fr vein)	7				3.375 ± 0.92
Maggio 2007	21	VA: femoral vessel<br>VV: R int jug + R common fem veins	19	8	2	0	
Hashiba 2012	12	Femoral a/v using Seldinger technique	12				2.0-5.0L/min/m2
Munakata 2012	10	Fem-fem	10				
Meneveau 2018	52		52				
Minakawa 2018	94		94				
Ius 2019	36	Femoro femoral, Femorofemoral and IJV	36				2-3 litres
Kjaergaard 2019	22	17Fr art<br>21Fr ven<br>femfem VA-ECMO	22				
Mandigers 2019	19		19				
Miyazaki 2019	9	fem-fem; (art: 16F, vein: 22F, superficial fem artery: 4F)	9				
Oh 2019	16	Femoro femoral	16				
Kaese 2020	12	femorofemoral 10 patients and femoral subclavian in 2 patients	12				
Sertic 2020	36	VA- FF VV- FIJV	32	18	4	2	4-6 litres
Giraud 2021	36	Femoral-femoral VA-ECMO: 36<br>Percutaneous VA-ECMO: 25	36				
Guliani 2021	17		17				
Goldberg 2021	27		27				
Hockstein 2021	17		17				
Lin 2021	13	FF	13				
Martinez-Solano 2021	14		14				
Prasad 2021	83	Arterial access site (RFA): 28<br>Arterial access site (LFA): 37<br>Venous access site (LFV): 26	83				

		Venous access site (RFV): 37<br>Venous access site (RIJ): 2					
Stadlbauer 2021	119		87		32		
Dumantepe 2022	29	fem-fem + distal perfusion cannula in superficial femoral artery	29	7			2-4L/min
Hobohm 2022	2197		1155	751	1042	436	
Jang 2022	9	16-18F arterial<br>20-22FR venous	9				
Mously 2022	9		9	2			
Danial 2023	41	Axillary: 2, Femoral: 38	41	20	0	0	
George 2023	10	Femoral: 9<br>right atrial to pulmonary artery cannula (Protek Duo) and connect to an ECMO Circuit: 1	9	4			
Kelty 2023	22		22	9			
Kucher 2023	10		10	5			
Sim 2023	15	Femoral: 15	15	6			

Studies not reporting relevant details have been removed from this table.

Abbreviations: ECMO: extracorporeal membrane oxygenation, VA: venoarterial, VV: venovenous

Table S4h. Subgroup demographics by region [9,10,26-62]

Subgroup		Studies	Mean	95% Confidence Interval
Age (years)	Europe	11	51.41	48.83 to 53.39
	Asia	11	57.59	54.90 to 60.29
	North America	10	50.19	50.19 to 55.63
Proportion of males (%)	Europe	14	59.73%	55.40% to 63.99%
	Asia	14	47.50%	37.79% to 57.29%
	North America	11	48.98%	41.62% to 56.37%
Pre-ECMO pH	Europe	6	7.07	6.94 to 7.20
	Asia	5	7.08	7.08 to 7.16
	North America	5	7.16	7.10 to 7.21
Pre-ECMO Lactate (mmol/L)	Europe	6	10.43	8.26 to 12.60
	Asia	2	12.53	11.59 to 13.47
	North America	6	6.87	4.94 to 8.81

Abbreviations: ECMO: extracorporeal membrane oxygenation

Table S5. Joanna Briggs Institute Checklist

Case series

Author	Domain										Total Score
	1	2	3	4	5	6	7	8	9	10	
Schmid 1991	?	✓	✓	✓	✓	✓	✓	✓	✓	✓	9
Kawahito 2000	?	✓	✓	?	?	✓	✓	✓	✓	✓	7
Maggio 2007	✓	✓	✓	?	✓	✓	✓	✓	✓	✓	9
Hashiba 2012	✓	✓	✓	✓	✓	✓	✓	✓	✓	✓	10
Munakata 2012	✓	✓	✓	✓	✓	✓	✓	✓	✓	✓	10
George 2018	✓	✓	✓	✓	✓	✓	✓	✓	✓	✓	10
Ius 2019	✓	✓	✓	✓	✓	?	✓	✓	?	NA	7
Kjaergaard 2019	✓	✓	✓	✓	✗	✓	✓	✓	✓	✓	9
Mandigers 2019	✓	✓	✓	✓	✓	✓	✓	✓	✓	NA	9
Oh 2019	✓	✓	✓	✓	✓	✓	✓	✓	✓	✓	10
Kaese 2020	✓	✓	✓	✓	?	?	✗	✓	✓	✓	7
Sertic 2020	✓	✓	✓	?	?	✓	✓	✓	✓	✓	8
Giraud 2021	✓	✓	✓	?	?	✓	✓	✓	✓	✓	8
Guliani 2021	✓	✓	✓	✓	?	✓	✓	✓	✓	✓	9
Hockstein 2021	✓	✓	✓	?	?	✓	✓	✓	✓	✓	8
Lin 2021	✓	✓	✓	?	?	✓	✓	✓	✓	✓	8
Martinez-Solano 2021	✓	✓	?	✓	✓	✓	✓	✓	✓	✓	9
Prasad 2021	✓	✓	✓	✓	✓	✓	✓	✓	✓	✓	10
Stadlbauer 2021	✓	✓	✓	✓	✓	✓	✓	✓	✓	✓	10
Dumantepe 2022	✓	✓	✓	✓	✓	✓	✓	✓	✓	✓	10
Jang 2022	✓	✓	✓	✓	✓	✓	✓	✓	✓	✓	10
Liu 2022	✓	✓	✓	✓	✓	?	✗	✓	✗	✓	7
Ltaief 2022	✓	✓	✓	✓	✓	✓	✓	✓	✓	✓	10
Mously 2022	✓	✓	✓	✓	✓	✓	✓	✓	✓	✓	10
Danial 2023	✓	✓	✓	✓	✓	✓	✗	✓	✗	✓	8
Kondo 2023	✓	✓	✓	✓	✓	?	?	✓	NA	✓	7

Legend:

✓: Yes (1 point)

✗: No (0 points)

?: Unsure (0 points)

NA: Not applicable (0 points)

Domains:

1: Were there clear criteria for inclusion in the case series?
2: Was the condition measured in a standard, reliable way for all participants included in the case series?
3: Were valid methods used for identification of the condition for all participants included in the case series?
4: Did the case series have consecutive inclusion of participants?
5: Did the case series have complete inclusion of participants?
6: Was there clear reporting of the demographics of the participants in the study?
7: Was there clear reporting of clinical information of the participants?
8: Were the outcomes or follow up results of cases clearly reported?
9: Was there clear reporting of the presenting site(s)/clinic(s) demographic information?
10: Was statistical analysis appropriate?

Cohort studies

Author	Domain											Total Score
	1	2	3	4	5	6	7	8	9	10	11	
Meneveau 2018	✓	✓	✓	✗	✗	✓	✓	✓	✓	NA	✓	8
Minakawa 2018	✓	✓	✓	✓	✗	✓	✓	✓	✓	NA	✓	9
Miyazaki 2019	✓	✓	✓	✓	✗	✓	✓	✓	✓	NA	✓	9
Goldberg 2021	✓	✓	✓	✗	✗	✓	✓	✓	✓	NA	✓	8
Hobohm 2022	✓	✓	✓	✓	✓	✓	✓	✓	✓	NA	✓	10
Panholzer 2022	✓	✓	✓	✗	✗	✓	✓	✓	✓	NA	?	7
Tsai 2022	✓	✓	✓	✓	?	✓	✓	✓	✓	NA	✓	9
Nasser 2023	?	✓	✓	?	✓	✓	✓	✓	✓	NA	?	7
Nishimoto 2023	✓	✓	✓	✓	✓	✓	✓	?	?	?	?	7
George 2023	✓	?	✓	✗	✗	✓	✓	✓	✓	NA	✓	7
Kelty 2023	✓	✓	✓	✗	✗	✓	✓	✓	✓	NA	✓	7
Kutcher 2023	✓	✓	✓	✗	✗	✓	✓	✓	?	?	✓	6
Sim 2023	✓	✓	✓	✗	✗	✓	✓	✓	✓	NA	✓	8

Legend:

✓: Yes (1 point)

✗: No (0 points)

?: Unsure (0 points)

NA: Not applicable (0 points)

Domains:

1: Were the two groups similar and recruited from the same population?
2: Were the exposures measured similarly to assign people to both exposed and unexposed groups?
3: Was the exposure measured in a valid and reliable way?
4: Were confounding factors identified?
5: Were strategies to deal with confounding factors stated?
6: Were the groups/participants free of the outcome at the start of the study (or at the moment of exposure)?
7: Were the outcomes measured in a valid and reliable way?
8: Was the follow up time reported and sufficient to be long enough for outcomes to occur?
9: Was follow up complete, and if not, were the reasons to loss to follow up described and explored?
10: Were strategies to address incomplete follow up utilized?
11: Was appropriate statistical analysis used?

Table S6. Grading of Recommendations, Assessment, Development, and Evaluations

№ of studies	Certainty assessment						Effect			Certainty	Importance
	Study design	Risk of bias	Inconsistency	Indirectness	Imprecision	Other considerations	№ of events	№ of individuals	Rate (95% CI)		
Mortality at closest time of follow up (assessed with: %)											
39	observational studies	not serious	not serious[a]	not serious	not serious	publication bias strongly suspected[b]	-	6409	mean 42.8% (37.2 to 48.7)	⊕⊕⊕◯ Moderate	CRITICAL
Duration of ECMO support (assessed with: days)											
26	observational studies	not serious	not serious[c]	not serious	not serious	none	-	1866	mean 4.3 days (3.6 to 5.1)	⊕⊕⊕◯ Moderate	IMPORTANT
Duration of intensive care unit stay (assessed with: days)											
17	observational studies	not serious	not serious[c]	not serious	not serious	none	-	1708	mean 12.6 days (9.7 to 15.4)	⊕⊕⊕◯ Moderate	IMPORTANT
Duration of hospital stay (assessed with: days)											
20	observational studies	not serious	not serious[a]	not serious	not serious	none	-	2753	mean 21.4 days (18.1 to 26.9)	⊕⊕⊕◯ Moderate	IMPORTANT
Duration of mechanical ventilation (assessed with: days)											
5	observational studies	not serious	Serious[d]	not serious	not serious	none	-	273	mean 7.2 days (2.7 to 11.6)	⊕⊕⊕◯ Moderate	IMPORTANT

Legend:

⊕◯◯◯: very low certainty level
⊕⊕◯◯: low certainty level
⊕⊕⊕◯: moderate certainty level
⊕⊕⊕⊕: high certainty level

Explanations

a. There is significant heterogeneity quantitatively and qualitatively. However, subgroup and regression analysis were able to elucidate covariates responsible for the heterogeneity
b. P_{egger}<0.0001. In view of the significant test from Egger's test, we downgraded the certainty for potential publication bias
c. There is significant quantitative heterogeneity. However, visual inspection of the forest plots shows that the point estimates are not sparsely distributed, and the confidence intervals overlap.
d. There is significant quantitative and qualitative heterogeneity.

Table S7. Summary of subgroup and meta-regression analysis

Subgroup analysis

Subgroup		Studies	Mortality	95% Confidence Interval
Region<br>$P_{interaction}$ = 0.0071	Europe; n=2618	14	53.1%	46.3% to 59.8%
	Asia; n=2737	14	37.8%	27.6% to 49.1%
	North America; n =1054	11	34.5%	25.6% to 44.6%
Study Type<br>$P_{interaction}$ = 0.8828	Case Series	26	42.7%	35.4% to 50.3%
	Cohort Studies	13	43.1%	34.0% to 52.7.%
VA vs VV<br>$P_{interaction}$ = 0.88	VA = 1963	34	42.37%	35.84% to 49.17%
	VV = 1048	3	41.82%	38.87% to 44.84%
Publication Year<br>$P_{interaction}$ = 0.3247	Before 2018	5	37.3%	27.1% to 48.9%
	2018 and after	34	43.6%	37.4% to 50.1%

Abbreviations: VA = venoarterial, VV = venovenous,

Meta-regression analysis

Covariate	Studies	B	LCI	UCI	P
Age (years)	32	-0.0426	-0.0984	0.0133	0.1353
**Prop. of patients with cardiac arrest**	**27**	**1.7719**	**0.2945**	**3.2493**	**0.0187**
Duration of ECMO (days)	26	0.0271	-0.1209	0.1751	0.7196
Prop. of males on ECMO	32	0.5457	-1.6262	2.7176	0.6224
Prop. of patients with a history of thrombosis	16	2.3110	-0.7934	5.4153	0.1446
Prop. of patients with prolonged immobilisation	14	0.2348	-2.2263	2.6960	0.8517
Prop. of patients having undergone recent operation	19	1.2519	-1.1778	3.6815	0.3126
Time to ECMO (hours)	7	0.0104	-0.1490	0.1697	0.8985
Prop. of patients with haemorrhagic complications while on systemic thrombolysis and ECMO	8	-0.0119	-6.9506	6.9267	0.9973
Prop. of patients with haemorrhagic complications while on ECMO	26	0.7697	-0.4702	2.0096	0.2237

Abbreviations: B: regression coefficient, ECMO: extracorporeal membrane oxygenation, LCI: lower confidence interval, P: P-value, UCI: upper confidence interval
Values in bold indicate p-value < 0.05.

Test for differences between definitive therapies with concomitant ECMO

Group	Group	P-val
**ECMO Alone**	**CDT**	0.0663
ECMO Alone	SE	0.7631
ECMO Alone	Thrombolysis	0.2545
**CDT**	**SE**	0.0872
**CDT**	**Thrombolysis**	**<0.0001**

SE	Thrombolysis	0.0621
SE + CDT	ECMO Alone	0.3153

Abbreviations: CDT: catheter-directed therapies, ECMO: extracorporeal membrane oxygenation, SE: surgical embolectomy. Values in bold indicate p-value < 0.05.

Further breakdown of catheter-directed therapy

Study	Patients receiving catheter-directed thrombolysis	Mortality of patients receiving catheter-directed thrombolysis	Patients receiving catheter-directed embolectomy	Mortality of patients receiving catheter-directed embolectomy
Sertic 2020	6	2	2	1
Giraud 2021	-	-	5	1
Jang 2022	-	-	1	1
George 2018	15	4	-	-
Lin 2021	4	1	-	-
Dumantepe 2022	29	7	-	-
Mously 2022	9	2	-	-

Studies not reporting specifically which catheter-directed therapy (embolectomy or thrombolysis) were excluded

Table S8: Summary of secondary outcomes

Outcome	Studies	Mean	95% CI	N
Duration of ECMO	26	4.4 days	3.6 to 5.1	1866
Length of ICU stay	17	12.6 days	9.7 to 15.4	1708
Length of hospital stay	20	22.5 days	18.1 to 26.9	2753
Duration of MV	5	7.2 days	2.7 to 11.6	273

Abbreviations: CI: confidence interval, ECMO: extracorporeal membrane oxygenation, MV: mechanical ventilation, ICU: intensive care unit

Table S9: Complications reported by included studies [9,10,26-62]

Author Year	Complications									
	Mechanical	Haemorrhagic	Neurological	Renal	Cardiovascular	Pulmonary	Infectious	Metabolic	Limb	Other
Schmid 1991			1 ischaemic brain damage		4 cardiac failure					
Kawahito 2000	Complications not reported									
Maggio 2007	3 Oxygenator Failure	8 Cannula Bleed<br>5 surgical site bleeding<br>1 Femoral Artery Pseudoaneurysm<br>1 HIT	5 stroke	5 creatinine >3.0mg/dL	12 haemodynamic instability requiring vasopressors<br>8 cardiac arrhythmias	3 pneumothorax	5 culture proven new infection<br>1 femoral cannula site soft tissue infection requiring debridement	4 hyperbilirubinaemia		
Hashiba 2012	Complications not reported									
Munakata 2012		10 cannula site bleed				1 alveolar haemorrhage<br>1 haemothorax				
Aso 2016	Complications not reported									
Corsi 2017		15 Hemorrhage	4 stroke	13 RRT			2 Surgical Site Infection		1 Limb Ischaemia	
George 2018		4 BGIT<br>5 Cannula bleed								
Meneveau 2018		90-day major bleeding: 20 ECMO vs 8 non-ECMO								
Minakawa 2018	Complications not reported									
Moon 2018		1 Pseudoaneurysm<br>4 Cannula bleed<br>1 Ulcer bleed	5 Hypoxic brain injury<br>2 neuropathy	7 AKI		2 Pulm haemorrhage<br>3 pneumonia	1 cannula site infection		1 compartment syndrome<br>2 limb ischemia	4 MOF
Elbadawi 2019	Complications not reported									
Ius 2019		2 Femoral Bleed		5 Renal		1 lung reperfusion edema	3 Infectious		1 Infectious	
Kjaergaard 2019	Complications not reported									

Mandigers 2019		14 ECMO Haemorrhage<br>1 Control Haemorrhage	2 ECMO Intracranial bleeding				5 ECMO Infectious complications			
Miyazaki 2019		1 puncture site pseudoaneurysm<br>1 puncture site haematoma	1 Neurological							
Oh 2019		9 Haemorrhage	1 Neurological						1 Arterial Ischaemia	
Kaese 2020	2 Mechanical	4 Haemorrhage							1 compartme nt syndrome	
Sertic 2020	12 Mechanical	14 Haemorrhagic	13 Neurological	11 Renal			3 Infectious		4 Limb	
Giraud 2021		ECMO only: 1<br>ECMO + thromblysis: 17	ECMO only: stroke (2) Anoxic encephalopathy (4)<br>ECMO + thromblysis: stroke (2) Anoxic encephalopathy (10)				ECMO only: 1<br>ECMO + thromblysis: 2			
Guliani 2021		1 vascular injury<br>5 bleeding requiring transfusion	1 stroke	1 AKI requiring dialysis						
Goldberg 2021		ECMO first: 0<br>SE only: 1 reop for bleeding<br><br>16 ECMO requiring blood transfusion<br>9 non-ECMO requiring transfusion	ECMO first: 1 stroke<br>SE only: 1 stroke	7 RRT ECMO<br>1 RRT nonECMO						
Hockstein 2021	4 distal perfusion cannula disoldgement	3 Arterial injury<br>1 Pseudoaneurysm<br>2 Haemorrhage	4 Stroke							3 visceral injury
Lin 2021		4 major bleeding								
Martinez-Solano 2021	Complications not reported									
Prasad 2021		Major bleeding in:<br>11 with systemic thrombolysis<br>17 without systemic thrombolysis								
Stadlbauer 2021	Complications not reported									
Stein 2021	Complications not reported									

Dumantepe 2022	2 surgical cannula-related wound-infection debridement	7 GUSTO<2 requiring blood transfusion	3 ischaemic stroke<br>1 hypoxic-ischaemic encephalopathy	4 AKI requiring RRT<br>2 requiring dialysis post-discharge						
Hobohm 2022			ECMO: 108 ICH<br>Control: 6953 ICH			1119 Pneumonia<br>517 ARDS<br>151 Pneumothorax<br>87 Haemopneumothorax				
Jang 2022		7 bleeding<br>1 pseudoaneurysm requiring surgical management<br>4 ECMO catheter insertion-site oozing								
Ltaief 2022		13 massive bleeding	7 anoxic encephalopathy	8 acute renal failure			4 Infectious			
Mously 2022	Complications not reported									
Tsai 2022		13 major bleeding	13 severe neurologic complications	11 severe kidney injury						8 ecmo-related complications

Abbreviations: ARDS: Acute respiratory distress syndrome, BGIT: Bleeding of the gastrointestinal tract, ECMO: Extracorporeal membrane oxygenation, HIT: Heparin-induced thrombocytopenia, ICH: Intracranial haemorrhage, MOF: Multiorgan failure, RRT: Renal replacement therapy
